# Mining for class-specific motifs in protein sequence classification

**DOI:** 10.1186/1471-2105-14-96

**Published:** 2013-03-15

**Authors:** Satish M Srinivasan, Suleyman Vural, Brian R King, Chittibabu Guda

**Affiliations:** 1 Department of Genetics, Cell Biology and Anatomy, University of Nebraska Medical Center, Omaha, NE, 68198-5145, USA; 2 Department of Computer Science, Bucknell University, One Dent Drive, Lewisburg, PA, 17837, USA; 3 Bioinformatics and Systems Biology Core, University of Nebraska Medical Center, Omaha, NE, 68198-5145, USA

**Keywords:** Discriminative *n*-grams, Class-specific motifs, Scoring function, *n*-gram model, Amino acid substitutions, Protein subcellular localization signals

## Abstract

**Background:**

In protein sequence classification, identification of the sequence motifs or *n*-grams that can precisely discriminate between classes is a more interesting scientific question than the classification itself. A number of classification methods aim at accurate classification but fail to explain which sequence features indeed contribute to the accuracy. We hypothesize that sequences in lower denominations (*n*-grams) can be used to explore the sequence landscape and to identify class-specific motifs that discriminate between classes during classification. Discriminative *n*-grams are short peptide sequences that are highly frequent in one class but are either minimally present or absent in other classes. In this study, we present a new substitution-based scoring function for identifying discriminative *n*-grams that are highly specific to a class.

**Results:**

We present a scoring function based on discriminative *n*-grams that can effectively discriminate between classes. The scoring function, initially, harvests the entire set of 4- to 8-grams from the protein sequences of different classes in the dataset. Similar *n*-grams of the same size are combined to form new *n-*grams, where the similarity is defined by positive amino acid substitution scores in the BLOSUM62 matrix. Substitution has resulted in a large increase in the number of discriminatory *n*-grams harvested. Due to the unbalanced nature of the dataset, the frequencies of the *n*-grams are normalized using a dampening factor, which gives more weightage to the *n*-grams that appear in fewer classes and vice-versa. After the *n*-grams are normalized, the scoring function identifies discriminative 4- to 8-grams for each class that are frequent enough to be above a selection threshold. By mapping these discriminative *n*-grams back to the protein sequences, we obtained contiguous *n*-grams that represent short class-specific motifs in protein sequences. Our method fared well compared to an existing motif finding method known as Wordspy. We have validated our enriched set of class-specific motifs against the functionally important motifs obtained from the NLSdb, Prosite and ELM databases. We demonstrate that this method is very generic; thus can be widely applied to detect class-specific motifs in many protein sequence classification tasks.

**Conclusion:**

The proposed scoring function and methodology is able to identify class-specific motifs using discriminative *n*-grams derived from the protein sequences. The implementation of amino acid substitution scores for similarity detection, and the dampening factor to normalize the unbalanced datasets have significant effect on the performance of the scoring function. Our multipronged validation tests demonstrate that this method can detect class-specific motifs from a wide variety of protein sequence classes with a potential application to detecting proteome-specific motifs of different organisms.

## Background

Most of the information pertinent to the structure and function of a protein is embedded in its primary structure, which is the long chain of amino acids. While the biological characteristics of a protein are a function of the sequence of amino acids it contains, various segments or the key peptides of proteins have specific roles in the overall protein’s function, and not all segments are equally important. These key peptides can differentiate a protein class from another in terms of their structure, function, subcellular location, phylogenetic class, etc. Hence, studying a protein sequence at the segment level (*n*-gram) has the advantage of understanding the functional components of a protein. In addition, *n*-gram-based approaches offer the computational advantage of expanding the search space for exhaustive comparison against other sequences. Popular bioinformatic algorithms such as BLAST have exploited this very concept to design algorithms for finding global and local similarities. These key peptides, referred henceforth as *n-*grams, have been widely used to identify homologous sequences, aligning sequences [1], clustering sequences [2], predicting subcellular localization [3], etc.

In protein sequence classification, it will be interesting to identify the sequence elements that can precisely discriminate between classes. In this study, we propose a new method to identify the discriminative *n*-grams, which are short peptide sequences that are highly frequent in one class but are either minimally present or absent in other classes. Our method implements amino acid substitution scores to detect not only the identical but also the similar *n*-grams, and a dampening factor to normalize *n*-gram scores across different classes with unbalanced datasets, resulting in the efficient detection of class-specific motifs.

Since the best equivalent of a word is not known in biological sequences, a short sequence of amino-acids *i.e.* an *n*-gram can be treated as a word, and statistical techniques can be applied on it to infer many interesting properties of the sequences. Using *n*-grams, statistical analyses such as determining zipf-like distributions and information theoretic measures such as perplexity can be performed to understand the frequency distribution of *n*-grams in the protein sequences [4]. *N*-gram models can also help in identifying sequence similarity, *n*-gram profiling [5], and in determining the conservation profile to identify protein homologs [6].

Using *n*-grams as features is a well-known technique in language processing and has recently been successfully applied in biological modeling. A comparative *n*-gram analysis on the entire genomes of 44 organisms has revealed that the frequent *n*-grams in one organism are also frequent in many organisms, and for each organism there is a small set of different *n*-grams that is specific to them [4]. Using these selective *n*-grams, obtained by chi-square feature selection method, they trained Bayesian classifiers and neural networks, which resulted in better protein family classification [4,7]. In other studies, the distribution of *n*-grams serves as a proteome-signature for organisms, which in turn can be used to determine evolutionary divergence at the genus level [8]. In this study, a 4-gram analysis across different organisms revealed that different organisms yielded different perplexity values. On considering only the top twenty 4-grams from *Neisseria,* most of the selective motifs in its protein sequences were successfully identified. It was also proposed that the relative abundance of specific *n*-gram types could be employed to study the species-specific properties of DNA modification, replication and repair mechanisms [8].

Selective *n*-grams have also been used for training SVM based classifiers [1]. It is hypothesized that the top-*n*-grams are the grams in which their constituent amino acids at any given position have a higher probability of occurrence at that position. Since these grams have a higher discriminative power to reveal most of the information about protein sequences, they are effective for remote homology detection and fold recognition [1]. Maetschke *et al.* (2010) have proposed an *n*-gram based conservation profile method that can be used for performing better sequence alignment by matching *n*-grams in linear time, compared to other well-known Smith-Waterman or Needleman-Wunsch methods, which have quadratic time complexity [5]. To exploit sequence homology, Leslie *et al.* (2004) have proposed the use of SVM’s to classify protein sequences in SCOP database based on functional and structural families. This method takes a discriminative approach by training SVMs with a special type of string kernels called mismatch kernels. For efficiently generating the matrix kernels *i.e.,* representative of the *k*-spectrum kernel denoted using positive weights for the *k*-mers with *m* mismatch instances, a (*k, m*) mismatch tree has been proposed. This tree is rooted up to the depth of *k*, where the leaf nodes are all possible *k*-mers in the dataset and each internal node with 20 branches represent the prefix of the leaf node *k*-mer. At each depth *d,* a depth-first search in this tree results in identifying valid instances of the *d*-length prefix of *k*-mers that are within *m* mismatches. On reaching a leaf node corresponding to a particular *k*-mer *α*, this method obtains pointers to all the instances of *k*-mers that are up to *m* mismatches from α. SVM’s trained with these mismatch kernels resulted in fast prediction of protein sequences *i.e.* in linear time and maintains good performance when training datasets are limited. In a subcellular localization study, a 7-gram model in conjunction with Bayesian classifier resulted in higher accuracy of prediction on the proteome sequences obtained from eight eukaryotic organisms [3]. Similar accuracy values are also observed on datasets from bacterial species using 6- and 8-grams [9].

Previously, we have developed a Bayesian supervised model for classifying protein sequence data using an *n*-gram approach [3], which can classify a set of sequences but cannot determine class-specific *n*-grams. In this study, we develop a novel scoring function that can effectively identify the discriminative class-specific *n*-grams from a given set of protein sequence classes, and validate our results using several datasets representing distinct biological functions. This method is compared against an existing method *Wordspy*[10], *mismatch string kernel* method presented in [11] and also applied against a new dataset to retrieve known Prosite patterns. Given the generic nature of this method, we believe that it can be used on a variety of protein sequence datasets to retrieve class-specific motifs.

## Results

### Scoring function

The scoring function proposed in this study for determining class-specific or discriminatory *n-*grams is based on an *n-*gram model. The scoring function is described at length in the methods section. In summary, it includes the following steps. First, *n*-grams of varying size are extracted from each class of protein sequences. Second, similar *n*-grams are identified using an amino acid substitution matrix (BLOSUM62) and their frequency counts are summed to obtain enriched counts. Third, a dampening factor is used to normalize the weights of *n*-grams from different unbalanced classes. Finally, a discriminative ratio (DR) is calculated for each *n*-gram to identify the class that contains this *n*-gram at least *T* times higher than the average of the second and third highest classes. Here, *T* represents a selection threshold at which DR is considered significant to be class-specific.

We applied our scoring function against the subcellular localization (SCL) dataset, which contains full-length protein sequences from 10 distinct subcellular locations. These data are experimentally-determined to exist in those locations (Table 1) and hence are used for training and testing our method. For validation, we report standard performance measures over each class, including sensitivity, specificity and AUC (area under the curve) using the ROC curves. (All of our formulae and definitions used in this study are briefly explained in the methods section). In addition to the standard validation tests, we also tested our results against experimentally known SCL signals to determine the effectiveness of the scoring function in terms of identifying class-specific motifs. This method is also applied against a completely different dataset to confirm if the method is able to recover class-specific patterns or not. For this purpose we used 50 different enzyme sequence families that have known Prosite patterns.

**Table 1:** Location-wise distribution of full-length and Pfam-mapped protein sequences

		**SL dataset**	**Pfam dataset**
**Organelle**	**Code**	**# of protein sequences**	**# of Pfam sequences**
Cytoskeleton	CSK	259	200
Cytoplasm	CYT	3334	2809
Endoplasmic Reticulum	END	1016	884
Extracellular	EXC	8666	6393
Golgi apparatus	GOL	291	248
Lysosome	LYS	159	138
Mitochrondria	MIT	2760	2383
Nuclear	NUC	5104	4221
Plasma membrane	PLA	6852	6155
Perixosome	POX	212	190

SL- Subcellular Localization; Pfam- Protein family database.

### Effect of amino acid substitutions on discriminatory *n*-grams

**Figure 1:**
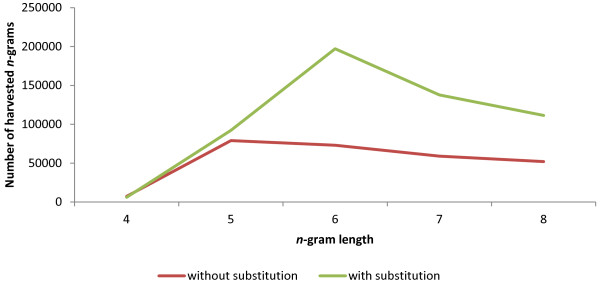
**Number of *n*-grams generated before and after substitution as a function of *n*-gram length at a selection threshold of 5.**

We used the scoring function on the SCL dataset to identify the entire set of discriminatory 4- to 8-grams. Any two *n*-grams of the same size that differ by a single amino acid at a single position are combined to form a new *n*-gram. This is done in accordance with the functionally equivalent amino acid substitutions based on the BLOSUM 62 amino acid substitution matrix (see Methods for more details). This technique yielded a significant increase in the number of *n-*grams available for analysis compared to the no substitution control. Figure 1 shows that at a selection threshold of 5 (*i.e.* the DR of each *n*-gram is greater than or equal to 5), the scoring function is able to harvest 5- to 8- grams at a rate of about 1.16 to 2.69 times (6-gram being the highest) more than without substitution. The increase in the number of *n-*grams harvested due to substitution can be attributed to the summation effect of the frequencies of similar *n*-grams. In contrast to the individual *n*-gram frequencies, the sum of the similar *n*-gram frequencies is sufficient to surpass the selection threshold, which makes the entire group of similar *n*-grams discriminant for a class.

### Evaluation of the performance of the method

We demonstrate the performance of the scoring function using specificity and sensitivity by mapping the discriminative *n-*grams back on to a derived dataset containing only the domain regions of the SCL dataset (mapped according to the Pfam domain database). A steady increase both in the sensitivity and specificity was observed with the increase in the selection threshold from 5 to 9 (Table 2). Even at a lower selection threshold of 5, the specificity values across all the classes were found to be above 96.1% and at the selection threshold of 10, the specificity attained a steady state *i.e.,* there was no significant change in the specificity values up to three decimal places. In contrast to specificity; at the selection threshold of 5 the sensitivity varied across classes from 73% to 98.7%. We observed a small drop in the sensitivity across eight of the ten classes at the selection threshold of 7 due to drastic reduction in the number of *n-*grams harvested at this selection threshold. Similar to specificity, the sensitivity also increased with increasing selection threshold. We notice that this behavior is interesting given the fact that sensitivity and specificity enhancements often have an inverse relationship. We reason that in this case, the size of *n*-gram space at each threshold is dynamic as against the conventional fixed size data space used for determining specificity and sensitivity. At higher score thresholds, we have less false negatives leading to higher sensitivity.

**Table 2:** Specificity and Sensitivity for different selection thresholds across different locations

**Subcellular location code**	**Specificity**						**Sensitivity**					
	**Selection threshold**						**Selection threshold**					
	**5**	**6**	**7**	**8**	**9**	**10**	**5**	**6**	**7**	**8**	**9**	**10**
CYT	0.974	0.98	0.979	0.983	0.985	0.985	0.865	0.895	0.888	0.907	0.921	0.912
CSK	0.997	0.998	0.997	0.998	0.998	0.998	0.73	0.771	0.769	0.801	0.826	0.822
END	0.985	0.988	0.987	0.989	0.99	0.99	0.93	0.95	0.951	0.962	0.97	0.97
EXC	0.973	0.979	0.979	0.982	0.985	0.985	0.899	0.924	0.923	0.939	0.949	0.95
GOL	0.996	0.997	0.996	0.997	0.997	0.997	0.983	0.996	0.994	0.998	0.999	0.998
LYS	0.998	0.998	0.998	0.999	0.999	0.999	0.987	0.993	0.987	0.993	0.997	0.988
MIT	0.982	0.985	0.984	0.987	0.988	0.988	0.877	0.898	0.879	0.895	0.906	0.900
NUC	0.977	0.981	0.98	0.984	0.986	0.986	0.749	0.807	0.806	0.844	0.871	0.871
PLA	0.961	0.97	0.968	0.974	0.978	0.977	0.878	0.897	0.893	0.908	0.919	0.919
POX	0.997	0.998	0.998	0.998	0.998	0.998	0.965	0.979	0.983	0.99	0.997	0.997

CYT – Cytoplasm; CSK – Cytoskeleton; END – Endoplasmic Reticulum; EXC – Extracellular/Secreted; GOL – Golgi; LYS – Lysosome; MIT – Mitochondria; NUC – Nuclear; PLA – Plasma membrane; POX – Perixosome.

**Figure 2:**
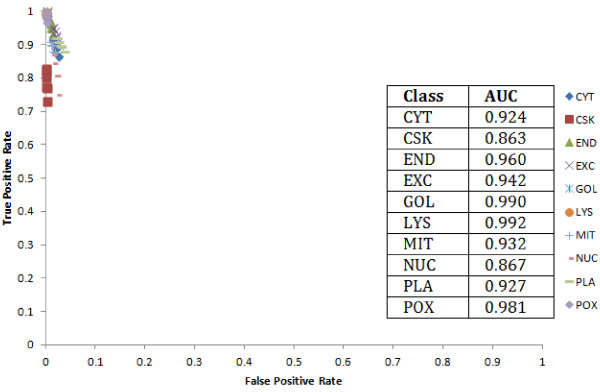
**ROC curve showing the performance of the scoring function in predicting true positive and false positive *n*-grams.** CYT – Cytoplasm; CSK – Cytoskeleton; END – Endoplasmic Reticulum; EXC – Extracellular/Secreted; GOL – Golgi; LYS – Lysosome; MIT – Mitochondria; NUC – Nuclear; PLA – Plasma membrane; POX – Perixosome.

We computed the false positive and true positive rates to plot the ROC curves (Figure 2). The ROC curves for our data points are all concentrated on the top left diagonal of the plot where the most number of true positives with the least number of false positives can be observed. We also determined the area under the curve (AUC) using the area under the trapezoidal method to get a quantitative measure of the ROC curve for each location. Locations with the highest AUC have the best performance. The smaller classes including POX, GOL and LYS have the best AUCs (0.981 to 0.992) indicating that the scoring function is efficient in identifying discriminatory *n*-grams pertaining to even smaller classes. For big to medium sized classes the AUC were found to be well above 0.863 and ranged from 0.863 (CSK) to 0.960 (END) indicating superior performance of the scoring function in identifying the discriminatory *n*-grams than any random guessing function.

### Comparison against Wordspy method

We compared the performance of our method with and without amino acid substitutions against a well-known discriminatory motif finder, *Wordspy*[10]. Wordspy was recommended as one of the best methods for discriminatory motif finding [12]. In this experiment, our goal is two-fold; first to evaluate the effect of amino acid substitutions over no substitution, and second to evaluate the performance of our method relative to Wordspy. In this section, we will denote our main scoring function that implemented the amino acid substitutions as SF1, and the scoring function without substitutions as SF2 for comparison.

**Figure 3:**
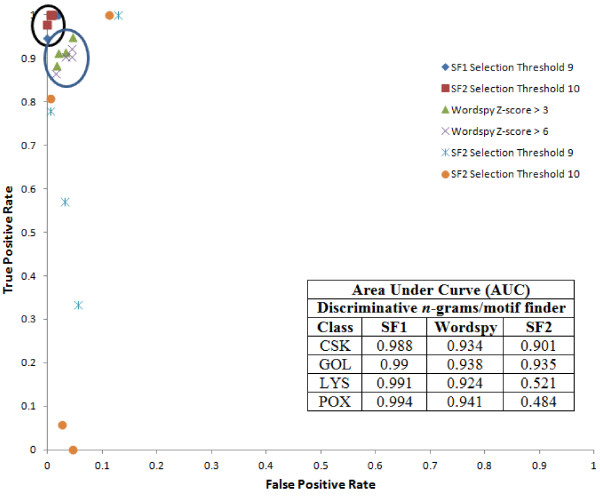
**ROC curve comparing the performance of SF1, Wordspy and SF2.** The black oval ring indicates ROC values of SF1 and the orange oval ring indicates ROC values of Wordspy. The black circle indicates the ROC region for SF1; the blue circle indicates the ROC region for Wordspy and the blue crosses and orange dots indicates the ROC region for SF2. CSK – Cytoskeleton; GOL – Golgi; LYS – Lysosome; POX – Perixosome.

The outputs of current method and the Wordspy are not directly comparable because Wordspy's output is Z-score based while our method outputs based on the DR ratio. Hence we used two data series for each method at higher stringency levels. In addition, we were able to conduct this experiment only for the classes with smaller datasets due to a limitation with the Wordspy in handling larger datasets (see Methods). We tested four smaller classes that include CSK, GOL, LYS and POX from the SCL dataset. Using SF1 and SF2 independently, we obtained 4- to 8-grams at selection thresholds of 9 and 10, and compared those with 4- to 6-grams (motifs) obtained from the Wordspy at the z-score thresholds of 3 and 6. A z-score threshold of 3 was suggested as the minimum default for a word in Wordspy [10]. We have tested z-score thresholds between 3–6 and found that the Wordspy’s performance stayed constant beyond the threshold of 4. Hence, we plotted the ROC curves only for thresholds 3 and 6. Outputs of the above three methods were independently mapped to the domain regions of corresponding class protein sequences to calculate true and false positive rates. Using these data, ROC curves were plotted and the AUC’s were determined to get a quantitative measure of the differences (Figure 3). We observed a superior performance of SF1 over Wordspy and SF2 across all the four classes as shown by the AUC metric. SF1 attained an AUC from 0.988 to 0.994, while AUC’s for Wordspy range from 0.924 to 0.941. On the other hand, AUC’s for SF2 ranged widely from 0.484 to 0.935. For CSK and GOL, all the three methods yielded a good AUC but for LYS and POX, SF1 and Wordspy’s performance was superior to SF2. Overall, our substitution-based method, SF1, performed better than the Wordspy and SF2. We want to emphasize that the difference in the performance between the scoring functions with (SF1) and without (SF2) amino acid substitutions is stark. This result also supports the need for using amino acid substitutions in this scoring function to efficiently recover discriminant n-grams.

### Comparison against mismatch string kernel method

The string kernels or mismatch kernels used by Leslie *et al.*[11] for training the SVM’s are very much similar to the *n*-grams harvested by our SF1, termed as *k*-mer or more precisely (*k, m*) *i.e., k*-mer with *m* (position) mismatches. These mismatch kernels help in capturing biological information about sequence similarity. The mismatch kernels work on the basis that any two protein sequences are similar if they share a large number of similar, and high positive weight mismatch kernels between them, under the assumption that mismatch kernels with high positive weights correspond to the conserved region in the protein families. Compared to the aforementioned mismatch kernels, the *n*-grams obtained from SF1 can be visualized as signals that can precisely discriminate protein sequences between classes.

Though *n*-grams and mismatch kernels are obtained by employing different methods, both of them essentially captures biological information from the protein sequences. Leslie *et al.*[11] have observed that their top 200 high-scoring *k*-mers obtained from (5, 1) mismatch kernel were within the nine-letter region of the alignment of five multiple alignment sequences in the SCOP dataset. For family-level homology detection, superfamily homology and for fold recognition in the SCOP dataset, they have showed that their discriminative SVM trained with (6,1) mismatch kernel performed better than the BLAST and Smith-Waterman [11]. On the other hand, our *n*-grams were promising to be class-specific signals as will be discussed more in the validation section below. Leslie *et al.* have indicated that their method suffers exponential time complexity for higher value of *m*[11]. Therefore they have considered *m*=1 for generating mismatch kernels which is similar to our substitution method *i.e.,* combining *n*-grams that are different from each other at any single position. Again both these discriminant methods have a low false positive rate.

### Computational complexity of the method

Our scoring function performs two major steps. It initially generates all the *n*-grams from protein sequences across different classes and creates a frequency profile for each *n*-gram. Secondly, it combines similar *n*-grams using the BLOSUM62 substitution matrix. For generating the *n*-grams and for creating the frequency profile from *M* number of sequences with *l* as the length of the longest sequence, across each class *c*_*t*_ the running time complexity can be gives as O(*cnMl*) where *n* is the length of the *n*-grams and *c* is the total number of classes in the dataset. For implementing substitution using the BLOSUM62 matrix our method breaks any given *n*-gram in to its constituent amino-acids and looks for each amino-acid substitution in a way that it is a valid substitution *i.e.* the entry in the BLOSUM62 matrix for that pair of amino-acid is greater than or equal to the BLOSUM62 threshold, and the new *n*-gram formed due to substitution of any single position change of amino-acid is present in the protein sequences of the dataset. Finally a new *n*-gram is created from the combination of all *n*-grams that differ from the original *n*-gram at any single position. In the worst case *i.e*. none of the *n*-grams are eligible for substitution, for *N* number of *n*-grams our method will have to consider all *N n*-grams and all *n* position. Thus the run time complexity for implementing substitution can be given as *O*(*NnO*(1)) or *O*(*nN*) where *O*(1) is the time required for searching the combining *n*-grams stored in a hash map. Therefore, the effective run time complexity of the method is *O*(*cnMl* + *nN*). For searching a substring or gram of length *n* in a protein sequence of length *l* our PERL-based scripts are implemented with run time complexity of *O*(*n*+log*l*).

### Validation of the discriminatory *n-*grams

Since discriminative *n-*grams are class-specific and striking features of a class, the scoring function should be able to identify these *n*-grams. We have validated the discriminative *n*-grams identified by our method against known SCL signals. This is accomplished by first generating the entire set of discriminant 4- to 8-grams from the protein sequences in the datasets, removing the *n-*grams that are sub-strings of other higher order *n-*grams, merging contiguous *n-*grams to obtain higher order *n*-grams that correspond to sequence motifs, and then looking for the known patterns in these motifs.

First, we tested our class-specific discriminatory *n*-grams against the nuclear localization signal database (NLSdb). The NLSdb is a comprehensive database containing information on nuclear localization signals (NLS), which are short stretches of residues that mediate transport of nuclear proteins into the nucleus [13]. We obtained discriminant 4- to 8-grams from each of the ten classes at a selection threshold of 9 (that corresponds to at least 87% sensitivity across all classes except for CSK) and performed two pruning steps namely removing the substrings and merging contiguous *n*-grams. When a pattern search was performed, our nuclear discriminant patterns matched with 128 out of 137 motifs from the NLSdb (found in NUC protein sequences within the SCL dataset), which is 93.4%. When the same search was performed using non-nuclear discriminant motifs, we could find only 33 NLS pattern matches (24%) in plasma membrane (PLA), and less than 3.6% of those in the other eight classes. This observation supports the fact that the scoring function is able to identify a rich set of class-specific functionally important motifs. The list of all the 137 NLSdb patterns is provided in the additional data file (Refer Additional file 1: Table S1). The NLSdb patterns are represented using the ‘RegEx’ language in PERL.

Since most of the extracellular (EXC) protein sequences are known to have signal peptides at the N-terminal region, we wanted to see if the scoring function is able to identify discriminatory *n*-grams from the N-terminal region of EXC protein sequences. We excised the first 50 amino-acid sequences of 1242 EXC proteins that were known to contain signal peptides at the N-terminal region [14]. By mapping the discriminative 4- to 8-grams of EXC, (obtained at a selection threshold of 9) we found that 82.4% of the 1242 EXC sequences had at least one discriminative 4- to 8-gram mapping to the N-terminal region. A similar experiment using discriminatory *n*-grams of other locations against their matched locations showed 45.2% and 31.2% for PLA and MIT locations, respectively, and very insignificant mapping for other locations. It is known that a large number of the plasma membrane (PLA) and mitochondrial (MIT) proteins harbor their targeting signals at their N-terminus [15,16]. The above results corroborate that those locations harboring targeting signals at the N-terminus are aptly identified by our method.

In addition to the subcellular targeting signals, we were also curious to check if the scoring function is able to identify other known functional motifs present on the protein sequences. Since our SCL dataset is classified based on the spatial distribution of proteins in the cell (as against functional family classification), we expect to see a wide distribution of functionally important sequence motifs across many locations. Using the ELM (Eukaryotic Linear Motif) database containing a rich set of non-globular functional linear motifs, we were able to validate a variety of patterns including nuclear localization signals (NLS), nuclear export signals (NES), post-translational modification signals (MOD), cleavage patterns (CLV) and ligand binding sites (LIG) at a selection threshold of 9. All the six nuclear (NES and NLS) signaling patterns listed in Additional file 1: Table S2.1 were recovered in the motifs obtained from nuclear location. Across the motifs (merged discriminative *n*-grams) of all the classes, we searched for the presence of 30 MOD patterns (listed in Additional file 1: Table S2.2) and identified 73% to 100% of them in different locations (Refer Additional file 1: Table S3.1 and S3.2). Similarly, we observed 86% to 100% of the CLV patterns (Refer Additional file 1: Table S2.3) in all but the LYS location, which showed only 71.4% of the CLV patterns. We also found the LIG patterns ranging from 55% to 97% in all but LYS location that has only 49% (Refer Additional file 1: Table S2.4). On an average, EXC and PLA motifs had all the three types (MOD, CLV, and LIG) of ELM patterns ranging from 97% to 98.9% (Refer Additional file 1: Table S3.1 and S3.2). It is possible that some of the smaller locations owing to their proteome size may not contain the entire cadre of proteins containing the above signals, which explains why the larger locations like EXC and PLA have most of the signals while the smaller locations like LYS have only a limited number of them. These observations strongly support our claim that the scoring function is able to identify both class-specific as well as functionally-significant motifs.

### Application of the method to recover Prosite patterns of enzyme families

**Figure 4:**
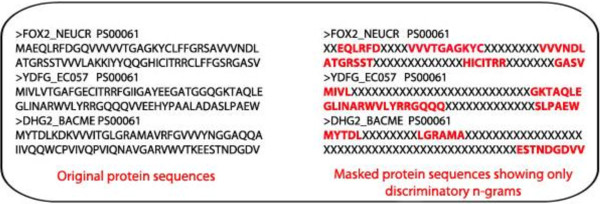
**Protein sequences with mapped discriminative *n*-grams and non-discriminative regions masked with ‘X’.**

**Figure 5:**
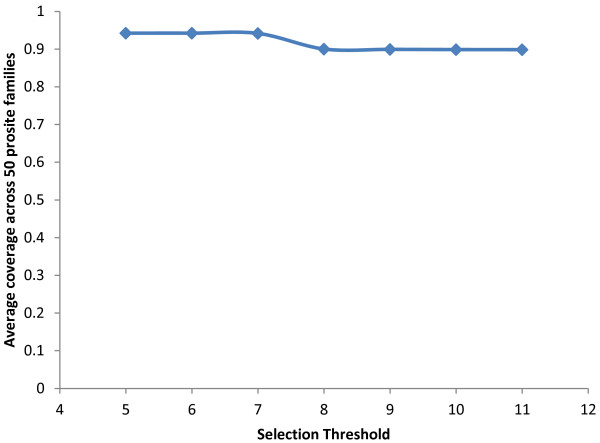
**Average coverage across 50 prosite families for different selection thresholds.** The blue line indicates the average coverage across 50 prosite families at different selection thresholds.

In this experiment our goal is to apply the scoring function against a different dataset and recover known patterns from the discriminatory *n*-grams. We have selected a dataset containing the Prosite domain regions of 50 enzyme families, where each family has a defined consensus Prosite pattern (Refer Additional file 1: Table S4). Using the scoring function, we generated discriminative 4- to 8-grams from the protein sequences in the Prosite dataset at varying selection thresholds from 5 to 11. The *n-*grams that were substrings of longer *n-*grams are removed and the resulting set of discriminant *n-*grams were mapped to the original Prosite domain sequences. After mapping the discriminant *n*-grams, the amino acid characters in the unmapped sequence regions are flipped to ‘X’ as shown in Figure 4; thus allowing only the discriminatory regions to exist in the sequences. Such masked sequences from each family are used to search against the Prosite pattern of corresponding family. We found that the average number of masked sequences containing the matched Prosite patterns ranged from 90% to 94.2% at selection thresholds of 11 and 5, respectively (Figure 5). At lower thresholds of 5–7, about 94% of the matches were found followed by a drop in the percentage of matches. Note that the average percentage of matches did not fall below 90% even at higher thresholds of 10 and 11 indicating that these highly conserved class-specific patterns are efficiently recovered by the scoring function even at higher thresholds. These results also demonstrate that this is a generic scoring function and hence can be used for classification and functional annotation of a variety of protein sequence classes.

## Discussion

We refer to the terms class and family in an interchangeable fashion because in the context of this paper, all families are classes but all classes are not families. For classification purpose, a class includes a set of identically labeled instances while a family includes a set of sequences with similar function. The datasets used in this study represent both classes and families. The SCL data, which represents classes, is based on the physical location of sorted proteins in the cell. Hence, these classes represent a heterogeneous mixture of proteins with different functions. In contrast, the PROSITE datasets are made of families, where each family represents a specific function. Hence *n*-grams from the SCL dataset are expected to be very diverse while those from the PROSITE are more homogeneous. Our results show that irrespective of these differences, the scoring function is able to discriminate between classes/families, which is anticipated to be more difficult with the SCL dataset.

The idea behind amino acid substitutions is to accommodate for changes in the amino acids by point mutations [5]. We considered only one amino acid change between a pair of *n*-grams when combining similar *n*-grams because in the length range of 4- to 8-grams, the chances of having more than one positively selected point mutation are very low. We used the amino acid substitution scores from the widely used substitution matrix, BLOSUM62 [17], which is adopted by the popular BLAST program as its default matrix [18]. Implementation of the amino acid substitution matrices to identify similar *n*-grams has considerably expanded the search space for exhaustive comparison of protein sequences thus enhancing the capability of the scoring function in detecting discriminant motifs (Figures 1 and 3).

The scoring function can generate *n-*grams of different sizes, but in this study we are focusing only on 4- to 8-grams. The reasons being; the search space of the distinct unitary, di- and tri-peptides is too small to explore the sequence landscape, while on the other end, the higher-order *n*-grams become less frequent, hence are too long to adequately explore the search space. A previous study showed that even with 4-grams most of the selective motifs in protein sequences are identifiable [8]. In our previous studies, we have optimized the *n*-gram length for similar datasets between 5–7 and we extended one order on either side in this study [3,9,19]. On the higher order, we were limited to generating only up to 8-grams because the scoring function suffered an exponential time complexity for generating *n*-grams and determining discriminatory *n-*grams beyond n=8. Despite this, we generated higher order discriminatory *n-*grams (*n* > 8) by combining different lower-order *n*-grams (4 ≤ *n* ≤ 8) if they overlap or are contiguous in the protein sequences of corresponding class.

The scoring function reports an *n-*gram as discriminatory if that *n-*gram is highly frequent in protein sequences of one class and is less frequent or absent in other classes. Therefore, it is ideal to have a balanced distribution of *n*-grams in the protein sequences of different classes. However, such distributions are difficult to achieve in biological datasets that are typically unbalanced and incomplete. Both the SCL and Pfam datasets used in this study are unbalanced in size across classes. To reduce the noise arising from this unbalanced distribution, we introduced a dampening factor into the scoring function. Multiplying the frequency count of each *n-*gram with this dampening factor results in giving a higher weightage to an *n-*gram that is observed in a single or fewer classes and a lower weightage to a *n-*gram that is distributed over many classes. We did not prefer to take into account the sequence count in each class (size) for normalizing the frequency count of an *n-*gram because, even in the balanced datasets, the natural frequency distribution of *n*-grams is not the same.

Based on the performance results (Figure 2), multipronged evaluation against known motifs and Prosite patterns (Figure 5) and comparison against another popular method, Wordspy (Figure 3), we conclude that our scoring function is more robust and can precisely discriminate between classes. It does not put any constraints on number of classes it can handle at a time for harvesting *n*-grams. In this work, we have considered the SCL dataset with ten classes and the Prosite dataset with fifty families. Secondly, the scoring function performs simple computation *i.e.* finding the frequency of each *n*-gram across all the classes, using a dampening factor to normalize its frequency across all classes. Finally, the scoring function has demonstrated better utilization of memory and reasonable execution time that increases exponentially with the increase of the *n*-gram size. In contrast, Wordspy performs sophisticated statistical calculations and engages in dictionary creation to discriminate motifs against background words. Our experience suggests that Wordspy has limitations with the size of datasets as well as with the size of the *n*-grams. Hence, to make a head-to-head comparison of the performance of our scoring function against Wordspy, we had to limit to using only four small classes (CSK, GOL, LYS and POX) from the SCL dataset, which in-turn suggests that Wordspy cannot be effectively exploited on larger datasets. In terms of the motif size, with Wordspy we could only work with 4- to 6-gram sizes and the program crashed on our 128 GB (RAM) machine due to memory explosion issues.

The method proposed here describes an alignment-free approach to identify class-specific regions in protein sequences. These class-specific regions are generally referred to as motifs, only if they are found in all the sequences of a particular family. We would like to point out that discovering the motifs is not the sole purpose but a byproduct of this work. Nevertheless, for validation purpose we used the known targeting signals from the subcellular locations or the patterns from the PROSITE database. The class-specific regions identified in this study include known motifs/signals as well as other contiguous regions of protein sequences that are highly enriched in a particular class or family of proteins. We hypothesize that the latter may represent potential unknown signals or otherwise functionally important class-specific regions of proteins. The functional importance of these highly enriched class-specific regions is worth investigating by experimental methods.

## Conclusion

Here, we have developed a scoring function to identify class-specific or discriminative *n*-grams for functional annotation of protein sequences. The scoring function implements BLOSUM62 based amino-acid substitutions and also normalizes the frequencies of the *n*-grams using a dampening factor to avoid noise due to their non-uniform distribution across different classes. At a selection threshold of 9, the scoring function identified a rich set of *n*-grams resulting in high specificity and sensitivity values across all the 10 subcellular locations. The scoring function also demonstrated a superior performance when compared against its variant (no substitution) and against a well known discriminative motif-finder, Wordspy. Validation against known subcellular localization signals and functionally-important motifs from the ELM database showed that the scoring function is able to identify most of those signals/motifs accurately. Application of the scoring function against a different dataset containing 50 enzyme families with known Prosite patterns resulted in the recovery of 90% of the sequences with correct patterns. These results clearly demonstrate that the scoring function is capable of discriminating the class-specific regions of protein sequence classes and is generic enough to be applied against a variety of sequence classes.

## Methods

### Datasets

Three different datasets were used in this study. The first one is the SCL dataset collected from the *Swiss-Prot database release 50.0*, which contains experimentally determined annotations on subcellular localization of proteins. The following filters were applied to obtain high quality data: only eukaryotic, non-plant sequences were considered; sequences with predicted or ambiguous localizations were removed; sequences shorter than 10 residues in length were removed; all redundant sequences were removed; and sequences known to localize in multiple locations were removed. The final SCL dataset consists of a set of 28,653 protein sequences distributed over 10 different organelles (Table 1). The second dataset contains the mapped domain regions of the first dataset, based on the Pfam domain database [20]. This mapping was done by cross-mapping the sequence IDs against the accession numbers in the *protein2ipr* database. Out of 28,653 sequences, only 23,621 proteins have at least one domain mapping against the Pfam-A domains. Table 1 provides the distribution of protein sequences in 10 different subcellular locations and corresponding Pfam mapped sequences. The third dataset was collected from the PROSITE database (Additional file 1: Table S4), which contains domain regions of the protein sequences from 50 enzyme families where each family has a known PROSITE pattern. The dataset consisted of a set of 26,863 protein sequences over 50 families. In the filtering step, it was ensured that there are no identical sequences within and across families. On average, there are 537 sequences in each PROSITE family used in this study.

### The *n*-gram model for protein representation

An *n*-gram is any subsequence of a protein of fixed length *n*. In literature, these protein subsequences have been called alternatively as *n*-grams, *n*-mers, *n*-peptides, etc. For the purpose of identifying discriminative *n*-grams, all possible *n*-grams are extracted from each protein sequence in the dataset. Given a dataset of protein sequences *D*, let *d*_*i*_ be a protein sequence in *D* where *d*_*i*_ = (s_1_s_2_…s_*k*_), where *s*_*i*_ ∈ Σ here Σ represent the set of all possible amino-acids, and |Σ| = 20 then a set of (*k*-*n*+1) grams can be obtained from *d*_*i*_*g*_1_ = (*s*_1_ … *s*_*n*_) *g*_2_ = (*s*_2_ … *s*_*n* + 1_), … *g*_*k* − *n* + 1_ = (*s*_*k* − *n* + 1_ … *s*_*k*_), Using the *n*-gram model, the following properties of *n*-grams can be observed.

• A discriminant gram *g*_*n*_ follows a Zipf-like distribution, *i.e.,* a discriminant gram is present *m* times, *m* > 1 more often in one class than the average of its frequencies in second and third highest class. Here *m is selection threshold also referred to as* DR.

• *g*_*n*_ is discriminatory in one class then it is not discriminatory in other classes.

For the following properties the observations are contingent based on the value of the selection threshold

• If a gram *g*_*n*_ is discriminatory in a class then all of its substrings *g*_*k*_ with length *k < n,* 4≤ *k* < 8 can be either discriminatory or non-discriminatory within the class and also across the classes. This property highlights the fact that the substrings of a discriminatory *n*-gram are not always discriminatory within and across classes. However, in our experiments we found few discriminatory *n*-grams whose substrings were also discriminatory within and across classes.

### Substitution of amino acids

We used the amino acid substitution matrix, BLOSUM62, as the reference matrix to identify functionally-similar and evolutionally accepted amino acid groups. A pair of amino acids with a positive substitution score (1 or more) is considered similar in our substitution scheme. Based on this, we combined fixed length similar *n*-grams that differ by a single amino-acid position. In the first step, the *n-*grams are combined irrespective of the class they belong to. Two *n-*grams, each with an amino-acid α and *β* at position *i ≤ n* are combined to form a new *n-*gram if the entry for (*α, β*) in the BLOSUM62 matrix is above or equal to a specified threshold (BLOSUM62 threshold). For example, consider a 6-gram *TLSNPK*. At a BLOSUM62 threshold of 1, *n-*grams such as *TLNNPK*, *TLSDPK*, *TLSNPK, TLSNPK* and *TLNSPK* can be combined to form a new 6-gram *TL*[*SN*][*NDS*]*PK*, where [*xy*] means either *x* or *y* will occur in that position. The assumption is that in the process of evolution, these amino acids replace each other in those positions by point mutations, yet underwent positive selection.

Since *n*-grams are subsequences of the protein sequences, each *n-*gram can have a different frequency count across different classes. The frequency count for a newly combined *n-*gram is the class-wise sum of the frequency counts of all its constituting similar *n*-grams. If the new *n-*gram *TL*[*SN*][*NDS*]*PK* is discriminatory to a class *C*_*1*_ then instead of *TL*[*SN*][*NDS*]*PK* the scoring function assigns all its constituting *n-*grams, *TLNNPK*, *TLSDPK*, *TLSNPK* and *TLSSPK* as discriminatory to the class *C*_*1*_ and updates the frequency count of each of these *n-*grams in class *C*_*1*_ equal to the frequency count of the combined *n-*gram *TL*[*SN*][*NDS*]*PK*.

### Scoring function

**Figure 6:**
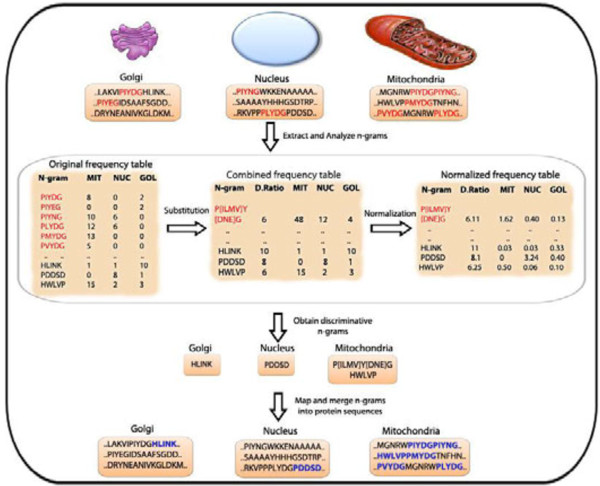
**A schematic diagram showing the methodology and scoring function.** GOL – Golgi; MIT – Mitochondria; NUC – Nuclear.

Figure 6 schematically represents the steps followed by the scoring function to determine discriminative *n*-grams and motifs from the protein sequences. The scoring function helps in determining a set of discriminatory *n*-grams based on the *n*-gram model discussed above. The scoring function is parameterized with the length of the *n-*gram, a selection threshold, and the target dataset to begin with. The scoring function reads in all the protein sequences for each class, and generates all possible *n-*grams for each sequence. If a protein sequence is of length *k* then the total number of *n-*grams is given by (*k-n + 1*). Once all the *n-*grams are generated, it implements amino acid substitution to combine a set of similar *n-*grams as described above in the substitution section. At the same time, the scoring function keeps track of the frequency counts of each *n-*gram (combined and single) across all the classes. In order to avoid noise due to the unbalanced nature of the dataset and also due to the non-uniform distribution of the occurrence of *n*-grams in nature, the scoring function normalizes the frequency counts of all the *n-*grams using a damping factor. This concept has been previously used in text mining literature as the TFIDF algorithm [21]. The dampening factor gives more weightage to the *n*-grams that are found only in a fewer number of classes as described below.

For an *n*-gram *x,* the damping factor is given by the expression $\ln\frac{\left|C\right|}{\left|\left\{c:x\in c\right\}\right|}$ where |*C*| denotes the total number of classes in the dataset and |{*c* : *x* ∈ *c*}| denotes the total number of classes in which the *n*-gram *x* is present. This damping factor is similar to the term ‘weighting’ as discussed in a previous study [22]. The scoring function multiplies the frequency count of each *n-*gram in a class with this damping factor. In some cases, it is possible for an *n*-gram to be present in all the classes thus making the expression $\frac{\left|C\right|}{\left|\left\{c:x\in c\right\}\right|}=1$ and consequently, the damping factor becomes 0, *i.e. *ln 1=0. To avoid this, we adjust the denominator expression as {*c*:*x*∈*c*}-0.1 i.e*.,* to have a larger numerator.

After determining the normalized frequency counts of all the *n-*grams in all the classes, the scoring function calculates a *discriminatory ratio* (DR) for each *n*-gram, which is determined as the ratio of its highest normalized frequency in a class to the average of its second and third highest normalized frequencies. If the DR is greater than the selection threshold, then the *n-*gram is considered as discriminatory to the class where the highest frequency (normalized) is observed. Variations in determining the DR is resolved using the following conditions:

• If the second highest and third highest normalized frequency values are 0, then the scoring function considers the mean value to be 1 to avoid divide by zero error.

• If the third highest normalized frequency value is 0, then the scoring function considers the mean value as equal to the second highest normalized frequency value.

• If the mean is between 0 and 1 then the scoring function considers the mean to be 1.

>The scoring function exploits the Zipf-like property of *n*-gram distribution in protein sequences across classes [4,8]. The scoring function picks an *n-*gram *z* as discriminatory for a class *y* if the frequency of occurrence of *z* in class *y* is greater than selection threshold times its average occurrence in any other two classes *v,w ≠ y*. For example, if a 5-gram *LMPQS* is discriminant in CYT at a selection threshold of 5 then it means that *LMPQS* appears in CYT more than 5 times the mean of its occurrence in other two highest classes. Due to substitution it is possible that the same discriminatory *n-*gram may appear in different classes with different DR. The scoring function filters the duplication of these discriminatory *n-*grams by retaining the *n-*gram in the class where its DR is the highest.

### Mapping and merging of *n-*grams

After obtaining a rich set of discriminative 4- to 8-grams the scoring function applies some filters on it. As mentioned in the *n*-gram model, it is possible that a sub-string of a discriminative *n-*gram could also be discriminative. For example a discriminative 5-gram, LMPQS can be a sub-string of a discriminative 7-gram, SLMPQST. The scoring function has a sub-string removal module implemented in PERL that identifies all the sub-strings (*n-*grams) and removes them from the list of discriminative *n-*grams while retaining the larger sized *n-*grams. This module initially sorts all the *n-*grams in an alphabetical order first and then based upon its size (lowest to highest). The sub-string removal function in this module performs a pair-wise comparison by taking a small sized *n-*gram as pattern and looks for it in larger sized *n-*grams. If a match is found the pattern is discarded and the larger sized *n-*gram is retained, else the pattern is retained.

Once the filtered set of discriminative 4- to 8-grams is available, the scoring function maps the discriminative *n-*grams of each class to the matched class sequences using a mapping and merging module. For each protein sequence in the class, this module creates a profile that includes the list of discriminative *n-*grams that can be mapped on to this sequence and their starting and ending position in the sequence. The profile for each protein sequence in the class is then sorted (ascending) based on the starting position of each discriminative *n-*gram. The module then merges the discriminatory *n-*grams *x* and *y* if the ending position of *x* is more, equal or one less than the starting position of *y* or vice-versa, to obtain contiguous higher order *n*-grams. Additional file 1: Table S5 demonstrates the steps performed by the mapping and merging module of the scoring function.

### Pattern matching with PERL regular expressions

We developed a PERL module in the scoring function to perform complex string comparisons using its regular expression patterns (RegExp) because PERL is well-known for being a flexible text-processing language. When a pattern is provided to this module, it scans across the list of discriminatory *n-*grams, motifs or merged discriminatory *n*-grams and/or protein sequences and reports whether that pattern exists or not. The patterns provided in well-known databases such as NLSdb, ELM and PROSITE do not follow the same convention as that of the PERL RegExp. Therefore, before the pattern matching step, we converted the native patterns to Perl RegEx patterns. For example, a NLSdb pattern *KKPx{6,9}Kx{1,3}RK* is equivalent to the Perl RegEx pattern *KKP.{6,9}K.{1,3}RK,* and a PROSITE pattern *G-S-x(2)-M-x-{RS}-K-x-N* is equivalent to PERL RegEx pattern *GS.{2}M.[^RS]K.N*. The PERL RegEx equivalents of NLSdb (Refer Additional file 1: Table S1) and PROSITE patterns used in this research are provided in the additional data file (Additional file 1: Table S4).

Let us consider the PERL RegEx pattern, *GS.{2}M.[^RS]K.N*, a pattern found in the *Fumarate_Lyases* (PS00163) family of sequences. In the above mentioned PERL RegEx pattern, the letters represent the amino acids, the ‘.’ indicates that any one of the twenty amino-acids can be substituted in that position, ‘{2}’ indicates any combination of two-letter amino acids that also includes repeat of same amino acid in both positions, and ‘[^*RS*]’ indicates any one amino acid that is not *R* and not *S*. The PERL-based pattern matching module can identify this pattern for the following sample of amino acid distributions in the PS00163 family protein sequences: XX*PENEP****GSSIMPGKVN****PTQC*XX, **GSSAMPYKRN**PMRSEXXX, XXEPFEKDQI**GSSAMPYKKN**, etc. (The bolded letters represent areas where there is a pattern match). The PERL module (s) are provided in Additional file 2.

### Performance metrics

We report standard performance measures over each class including the following: *true positives* (TP) as the number of *n-*grams that are correctly identified in a class that belongs to them; *false negatives* (FN) as the number of *n-*grams that are not identified in a class that belongs to them; *true negatives* (TN) as the number of *n-*grams that are not found in a class that doesn’t belong to them; *false positives* (FP) as the number of *n-*grams that are identified in a class that doesn’t belong to them; *specificity* can be observed as a ratio of TN to the sum of TN and FP; and *sensitivity* as the ratio of TP to the sum of TP and FN. We used a confusion matrix to keep track of all these values, where, the entries in the diagonal represent the TP for each class; the sum of the entries in each row minus the TP is FN; the sum of the entries in each column minus the TP is FP, and the sum of the rest of the entries in the confusion matrix is TN.

We also report coverage in a class as the ratio of number of sequences in which a pattern match was found to the number of sequences in that class. A pattern match occurs only when an *n-*gram satisfying the rules (PERL RegEx) described in the pattern exists.

### Generating motifs using Wordspy program

We downloaded the Linux executables (binaries) for Wordspy on a 48-core 2.00 Gz machine with 128 GB RAM. Using the command line option, we supplied Wordspy with a positive data set and a negative data set containing protein sequences in FASTA format. Datasets belonging to only four smaller classes that include CSK, GOL, LYS and POX were used in this experiment. We created a positive data set file including protein sequences of one class and a negative data set file containing protein sequences from the rest of the three classes. For example, if the positive data set included protein sequences from CSK then the negative data set included protein sequences from GOL, LYS and POX. This step was repeated for each of the four classes. We instructed Wordspy to identify all significant 4- to 6-gram motifs, each with a z-score greater than 3 and 4. In addition to that Wordspy was commanded to identify motifs that occurred more than 5 times in the protein sequences of positive data set. Once we obtained the motifs from Wordspy we supplied it to our pipeline for obtaining ROC curves and determined AUC’s for each class.

## Abbreviations

AUC: Area under curve; BLAST: Basic local alignment search tool; BLOSUM62: BLOcks of Amino Acid SUbstitution Matrix; CLV: Clevage site; DNA: Deoxyribonucleic acid; DR: Discriminative ratio; FASTA: Fast A; ELM: Eukaryotic linear motif; FN: False negative; FP: False positive; LIG: Ligand binding site; MOD: Post-translational modification; NES: Nuclear export signals; NLS: Nuclear localization signals; NLSdb: Nuclear localization signal database; PERL: Practical extraction and reporting language; Pfam: Protein family; RAM: Random-access memory; RegExp: Regular expression; ROC: Receiver operating characteristic; SCL: Subcellular localization; SF1: Scoring function with substitution; SF2: Scoring function without substitution; SVM: Support vector machine; TN: True negative; TP: True positive; TRG: Mitochondrial import signals.

## Competing interests

The authors declare that they have no competing interest.

## Authors’ contributions

SMS developed the scoring function, carried out the analyses and drafted the manuscript. SV developed some core modules of the scoring function pipeline in python and assisted in the application of the method to enzyme family datasets. BRK has written the original ngLOC code, which is used as a basis for the scoring function. CG conceived the original study, generated the datasets, conceptually provided the framework for the scoring function and for the project and assisted in the manuscript preparation. All authors have read and approved the final manuscript.

## Authors’ information

SMS is a Postdoctoral Research Associate with a strong background in computer science. SV is a graduate student with training in computer science. BRK (Assistant professor) has a strong background in computer science and mathematics. He was a former student in CG’s group and co-authored the ngLOC method with CG. CG (Associate professor) has an interdisciplinary background in molecular and computational biology. He has published a number of computational methods with a variety of applications in biomedical research, since 2001.

## Supplementary Material

Additional file 1: Table S1 List of 137 NLSdb patterns encoded by Perl RegEx patterns. **Table S2.1.** ELM Perl RegEx patterns for Nuclear Export Signals (NES). **Table S2.2.** ELM Perl RegEx patterns for Post-Translational Modification Sites (MOD). **Table S2.3.** ELM Perl RegEx patterns for Clevage Sites (CLV). **Table S2.4.** ELM Perl RegEx patterns for Ligand Binding Sites (LIG). **Table S3.1.** ELM patterns observed in the protein sequences of Subcellular Localization dataset. **Table S3.2.** ELM patterns observed in the motifs obtained from subcellular localization dataset at a selection threshold of 9. **Table S4.** Prosite patterns of 50 enzyme families. **Table S5.** Mapping and merging of discriminative *n-*grams. Click here for file

Additional file 2 **This file contains source code for six PERL modules used in this project.** A full description of each program, usage and source code are provided in the README section at the beginning of the file. Click here for file
