# Supplementary material for: Mining for class-specific motifs in protein sequence classification
Source: BMC Bioinformatics. 2013 Mar 15;14:96. doi: 10.1186/1471-2105-14-96 (PMC3610217; doi:10.1186/1471-2105-14-96)
Supplement: Additional file 1: Table S1 — List of 137 NLSdb patterns encoded by Perl RegEx patterns. Table S2.1. ELM Perl RegEx patterns for Nuclear Export Signals (NES). Table S2.2. ELM Perl RegEx patterns for Post-Translational Modification Sites (MOD). Table S2.3. ELM Perl RegEx patterns for Clevage Sites (CLV). Table S2.4. ELM Perl RegEx patterns for Ligand Binding Sites (LIG). Table S3.1. ELM patterns observed in the protein sequences of Subcellular Localization dataset. Table S3.2. ELM patterns observed in the motifs obtained from subcellular localization dataset at a selection threshold of 9. Table S4. Prosite patterns of 50 enzyme families. Table S5. Mapping and merging of discriminative n-grams. [file 1471-2105-14-96-S1.docx]

**Supplementary information for Srinivasan et al.**

**Table S1**: List of 137 NLSdb patterns encoded by PERL RegEx patterns

| 1. PLLKKIKQ 2. PPQKKIKS 3. K[RK]{3,5}.{11,18}[RK]K.{2,3}K 4. PAAKRVKLD 5. RRSMKRK 6. RRMKWKK 7. RKRKK 8. RKRRR 9. RPRRK 10. RRERNKMAAAKCRNRRR 11. [KR]{4}.{20,24}K{1,4}.K 12. KRMRNRIAASKCRKRKL 13. KRAAEDDEDDDVDTKKQK 14. KRSAEGGNPPKPLKKLR 15. [DE]K[NIF]RR[DEK][STMNQ] 16. [DE]KR[MQN]R[MQN]R 17. R[RK].{4,6}[RK][RK].[RK].{1,3}[RK][RK][PLQ] 18. R[RK]{2,4}[PL][RK][MNQ]R 19. R[RK]{2,4}.{15,19}[RK]{2,4}[QLM]K 20. R[RK]{3,}?[DE]K 21. [DE]K.RRK[MNQ] 22. [DE]RKRR[DEPLQ] 23. [DE]R.KKKK 24. R[PL]..[KR]{2,}?..[KR]V 25. [DE][KR]RR[KR][FYW] 26. R[QMPL]RR[DE]R 27. R[RK]K[RK]KR 28. R[RK].[KR].[RK]{2,}?[DE] 29. R[STCMNQ]R[STCMNQ]KR 30. R{2,}?PR{3,}? 31. D[KR].{0,1}[QL][RK]{2,3}R 32. R{2,3}K{3,4}[PLRKE] 33. R.{2,3}RRRRRR 34. R.{2,3}H.{3,5}RRRR 35. R.[KR][KR]K[PLQM]R 36. R.R{2,}?[QL].[ST]R 37. R.R.R.R.R.R 38. R.RSRS.{0,1}R.R 39. DK[QL]KK[QL] 40. R.RR.{4,6}RKK 41. R{2,}?[QMN]R{3,}? 42. [QL].KR.K.KK 43. [QL]K{2,4}.{8,12}[RK][QL][RK][QL]KR 44. [PL]..KR[IV]K[PL][DE] 45. [PL][RK][RK][KR][GAPL][RK][STQM] 46. [PL][RK][RK][DEP]R[RK][FYW] 47. [PL][KR]{5,7}[PL] 48. [RK]H[RK]...[RK]{2,4}.R 49. [RK]R[MS]K.K[KR] 50. [STQM]RRRK[STQM] 51. [STQM]RKRR[STQM] 52. [STQM]RKRK[STQM] 53. [RK]{3,}?.{8,16}[RK]{4,}? 54. [RK]{3,}?.[RK].[RK].{4,9}[RK]{3,}? 55. [RK]{2,4}.{2,4}[QLM][RK].{2,3}[RK]KR 56. [RK]{2,4}.{1,2}[RK].{0,2}[RK].{3,5}[RK].{0,2}[RK][RK]{2,4}[PL] 57. [RK].[RK].[KR].{4,6}RKK 58. [RK][PLIV][KR][RK]{2,4}[PLVI]R 59. [PL]K..KRR 60. [PLV]RK[ST]R[DE]K 61. [KR]KRKK 62. [QMN]R[RK].K.[RK][RK] 63. [GA][KR]KR.[KR][GA] 64. [GA]R.[RK].[RK][RK].[QM] 65. [ED]R{4,}?[ED] 66. [DE][ST][PL]KR[STC] 67. [KR][KR][KR][KR][KR][KR][KR] 68. [KR][KR].[KR][KR][KR].[KR][KR] 69. [PLV]K[RK].[RK][RK][RK][PL] 70. [PLV]K[RK].[QMN][RK]R 71. [PLQ][KR].{3,4}KKRK 72. [PLQ]K[RK].{1,2}[RK].{3,6}[RK][RK].{1,2}[RK].{1,2}[RK][RK] 73. [PLQMNKR]K[KR][KR]R.K[PLQMNKR] 74. [KR]{2}.{0,1}[KR]{2,4}.{25,34}K{2,4}.{1,2}K 75. [KR]{2,3}..KR[KR][QLM] 76. [DE][RK]{3,}?.[KR]{2,}?[PL] 77. KRK.{5,10}KK[PL]K 78. LKKIKQ 79. KR[GPL]R[GPL]R[GLP]RK 80. KR[MNSQ]R[MNSQ]R 81. KK.{1,7}K[PL][PLIV]KK 82. RER[MNQ]K.{4,8}R[MNQ]RR 83. KKRKR[KR] 84. Q[RK][HRK][RK].RR 85. KKRRK 86. P.[PQLVMN][KR]{2,3}.KQ 87. KKRR.K 88. KK[MNQSTC]R[MNQSTC]K[MNQSTC] 89. K{3,4}R{2,3} 90. K[GA]K[AG]KK[AG] 91. KR[RK][RK].{2,4}[RK].{0,2}R.{3,5}[RK].{0,2}[RK].{0,2}[RK][RK]K 92. KR[ST]R..R{2,4}[QL]K 93. KR.R.R.{2,6}RKRK 94. KR.R..RRLK 95. KR{3,}?[LVI] 96. KR{2,4}.{3,6}[RK]{2,4}.{0,2}KR 97. KR.[DE][KR][KR].K 98. K[KR][KR]RR[KR] 99. K.[PLV][RK][RK]RK 100. K.K.K.....RKK 101. K[RK]{2,}?[QL].{3,8}R{3} 102. K[PL]K{2,3}.{1,3}[RK]{2,4}.{6,9}K[KR] 103. KR.{1,3}H.{3,5}R[LQ]RR 104. R[PL].G.[KR][KR].K 105. RR.RRRRR 106. RR.KR.K[PLV] 107. RR[TS].[QK][KR][KN] 108. RR[TS].[QK][KR][KNS] 109. RR[PLQMN].RRRR 110. RR[PLIV]RK.K 111. RRR{3,5}T 112. RR..KRK 113. RR.{0,1}RRRRR 114. R[MNQ].{4,8}R[MNQ]RR 115. R[KR]{3,4}K[DE] 116. R[KR][RK].{0,2}[RK].{0,2}[RK].{3,5}[RK].{0,2}[RK][RK][RK][RK][PMQL] 117. R[KR]RRRR.R 118. R[IVLP][IVLP]KRR 119. R[GVLIP]RRRR.R 120. GR[RK]{2,4}..[RK][QL] 121. RRR[PL]RK 122. RRRRR.RR 123. RKR{3,5}[ST] 124. RKR.{12,16}RRKK 125. RKR[PLQMN]R[PLQMN]R 126. KKKKK.{3,6}KK 127. KKKKR[KR] 128. RKKRKR 129. KKP.{6,9}K.{1,3}RK 130. RK[PL][PLV]KK[RKH] 131. RK[RK][QML][RK].R 132. RRRRRR.{0,2}R 133. RRRRRR 134. RRK.{5,7}RRR 135. RRK.{3,5}R[DE]R{3,}?[PLV] 136. RRER[MNQ]K.{4,8}R[MNQ]RRR 137. RH[RK]H.{2,4}[RK]{2,4}[PL]R |
| --- |

**Table S2.1**: ELM PERL RegEx patterns for Nuclear Export Signals (NES) & Nuclear Localization Signals (NLS)

| ELM Identifier | **RegEx** |
| --- | --- |
| TRG_NES_CRM1_1 | [DEQ].{0,1}[LIM].{2,3}[LIVMF][^P]{2,3}[LMVF].[LMIV].{0,3}[DE] |
| TRG_NES_CRM1_1 | [DE].{0,1}[LIM].{2,3}[LIVMF][^P]{2,3}[LMVF].[LMIV].{0,3}[DEQ] |
| TRG_NLS_Bipartite_1 | [KR][KR].{7,15}[^DE]((K[RK])\|(RK))(([^DE][KR])\|([KR][^DE]))[^DE] |
| TRG_NLS_MonoCore_2 | [^DE]((K[RK])\|(RK))[KRP][KR][^DE] |
| TRG_NLS_MonoExtC_3 | [^DE]((K[RK])\|(RK))(([^DE][KR])\|([KR][^DE]))(([PKR])\|([^DE][DE])) |
| TRG_NLS_MonoExtN_4 | (([PKR].{0,1}[^DE])\|([PKR]))((K[RK])\|(RK))(([^DE][KR])\|([KR][^DE]))[^DE] |

**Table S2.2**: ELM PERL RegEx patterns for Post-Translational Modification Sites (MOD)

| 1. C.([DN]).{4,4}[FY].C.C 2. (C)[^DENQ][LIVM].$ 3. ...([ST])P.[KR] 4. S..([ST])... 5. ...([ST])..E 6. (W)..W 7. (.)G[RK][RK] 8. [ED]{0,3}.(S)[GA]. 9. ...([ST])...[ST] 10. .(N)[^P][ST].. 11. (N)[^P]C 12. ^M{0,1}(G)[^EDRKHPFYW]..[STAGCN][^P] 13. C.{3,5}([ST])C 14. C.(S).PC 15. ...([ST])Q.. 16. [RK]..(S)[VI].. 17. [RK][RK].([ST])[^P].. 18. .R.([ST])[^P].. 19. R.R..([ST])[^P].. 20. .[DE].([ST])[ILFWMVA].. 21. ...([ST])P.. 22. G(C)M[GS][CL][KP]C 23. ^M{0,1}G(C)..S[AKS] 24. [VILMAFP](K).E 25. [TAD][EA].Q(Y)[QE].[GQA][PEDLS] 26. ..[RKTC][IVL]Y[TQHS](Y)[IL]QSR 27. [DE]..(Y)..[LI].{6,12}(Y)..[LI] 28. [ILV].(Y)..[ILV] 29. ..T.(Y)..[IV] 30. [ETA](C)[QERK]..F...RWNC[ST] |
| --- |

**Table S2.3**: ELM PERL RegEx patterns for Cleavage Sites (CLV)

| 1. [ILV]..[R][VF][GS]. 2. (.RK)\|(RR[^KR]) 3. R.[RK]R. 4. [KR]R. 5. KR. 6. [R]...[KR]R. 7. [RK].[AILMFV][LTKF]. 8. Q[MLVI]DG..[DE] |
| --- |

**Table S2.4**: ELM PERL RegEx patterns for Ligand Binding Sites (LIG)

| 1. R.[^P]([ST])[^P]P 2. R..[^P]([ST])[IVLM]. 3. [RHK][STALV].([ST]).[PESRDIF] 4. [IL]..[^P][^P][^P][^P]R.....[IL]..[^P][^P][ILV][ILM] 5. [R]..[ILVMF][ILMVF][^P][^P][ILVM].{4,7}L(([KR].)\|(NK))[VATI] 6. [^R]..((.[ILMVF])\|([ILMVF].))[^P][^P][ILVM].{4,7}L(([KR].)\|(NK))[VATIGS] 7. F..[FWY][ST][FY] 8. F..[FWY][DE][FY] 9. F..F$ 10. F.D.F 11. DP[FW] 12. .R..L..[LIVM]. 13. .KEN. 14. [DE][DES][DEGAS]F[SGAD][DEAP][LVIMFD] 15. ^M{0,1}[AS]... 16. ^M{0,1}A.P. 17. DA.P. 18. ^M{0,1}A.[AP]. 19. DA.G. 20. .(S)..F 21. .(S)..F.K 22. .(S)..Y$ 23. [ED].{0,2}[ED].{0,2}[EDQ].{0,1}[YF]$ 24. L[IVLMF].[IVLMF][DE] 25. .[np]w[des].w 26. [DE][DE]...VP[DE] 27. L[^P]{2,2}[HI]I[^P]{2,2}[IAV][IL] 28. [PG][LVIPME][DENS]L[VASTRGE] 29. [RK].L.{0,1}[FYLIVMP] 30. [^P].[KR].TQT 31. .NPF. 32. .[FYH].[IVM][^WFYP][^WFYP][ILM][ILMV]. 33. [FILVY].{0,1}P.[PAILSK]P 34. PP..F 35. [FY].[FW].....[LMVIF]P.P[DE] 36. [LV][DE][^P][LM][LM][^P][^P]L[^P] 37. ..(T)..[ILV]. 38. ..(T)..[DE]. 39. [EN][FYLW][NSQ].EE[ILMVF][^P][LIVMFA] 40. [QHR].{0,1}P[PL]PP[GS]H[RH] 41. [DE]H.Y 42. [FY][DEP]WM 43. P[MVLIRWY]V[MVLIAS][LM] 44. G[FL]PGER..G 45. ...[SACLIVTM]..[ILVMFCT]Q.{3,3}[RK].{4,5}[RKQ].. 46. [VILMFT]K.EP.[DE] 47. [VILMFT]K.EP.{2,3}[DE] 48. [VILMFT]K.EP....[DE] 49. [LM]YP...[LI][^P][^P][LI] 50. [LM]YP.[LI] 51. [KR][IV][LV].....P 52. [KR]{0,2}[KR].{0,2}[KR].{2,4}[ILVM].[ILVF] 53. F.FP 54. F...W..[LIV] 55. [^WFHYG][MAPSTLIV]P[^CGW]L[^EDKRPG]P[^WFHYG] 56. [^P]L[^P][^P]LL[^P] 57. [IL]A(P).{6,8}[FLIVM].[FLIVM] 58. ..[LFP][NS][PIVTAFL].A..(([FY].[PYLF])\|(W..)). 59. ((^.{0,3})\|(Q)).[^FHWY][ILM][^P][^FHILVWYP][DHFM][FMY].. 60. ...[ST].[ACVILF]$ 61. ...[VLIFY].[ACVILF]$ 62. ...[DE].[ACVILF]$ 63. ..[RK].{0,1}[VI][^P][FW]. 64. .P[^P]I[^P][IV][^P] 65. .P[TS]AP. 66. (.[^P].NP.[FY].)\|(.[ILVMFY].N..[FY].) 67. (.[^P].NP.(Y))\|(.[ILVMFY].N..(Y)) 68. F[EDQS][MILV][ED][MILV]((.{0,1}[ED])\|($)) 69. [LI].C.[DE] 70. ..[LIMV]..[LM][FY]D. 71. RGD 72. .[ILVM]LG..P. 73. [LIVMP].{0,2}(T)P..([ST]) 74. [LIVMP].{0,2}(T)P..E 75. D(S)G.{2,3}([ST]) 76. (Y).N. 77. (Y)[IV].[VILP] 78. (Y)[QDEVAIL][DENPYHI][IPVGAHS] 79. (Y)..Q 80. (Y)[VLTFIC].. 81. G(Y)[KQ].F 82. [RKY]..P..P 83. P..P.[KR] 84. ...[PV]..P 85. KP..[QK]... 86. P..DY 87. .P.A.V.P[^P] 88. [LIV]..[LM]L.AA.[FY][LI] 89. [FHYM].A[AV].[VAC]L[MV].[MI] 90. [FA].[LA][LV][LVI]..[AM] 91. RF[^P][IV]. 92. ([KR][^ED]{0,5}[ST].IP[^ED]{5,5})\|([^ED]{5,5}[ST].IP[^ED]{0,5}[KR]) 93. R..[PA]DG 94. EEVD$ 95. [PSAT].[QE]E 96. P.Q..D 97. ..P.E..[FYWHDE]. 98. [FY].L.P 99. [KR]{1,4}[KR].[KR]W. 100. [PA][^P][^FYWIL]S[^P] 101. P.E[^P].S[^P] 102. [WFY]RP[WFY].{0,7}$ 103. [WFY][KR]P[WFY] 104. PP.Y 105. PPLP 106. .PPR. 107. ...([ST])P. |
| --- |

**Table S3.1**: ELM patterns observed in the protein sequences of Subcellular Localization dataset

| Class | CLV | LIG | MOD |
| --- | --- | --- | --- |
| CYT | 8 | 98 | 24 |
| CSK | 7 | 77 | 23 |
| END | 7 | 96 | 23 |
| EXC | 7 | 99 | 26 |
| GOL | 7 | 86 | 24 |
| LYS | 7 | 71 | 22 |
| MIT | 8 | 94 | 23 |
| NUC | 8 | 99 | 24 |
| PLA | 8 | 98 | 25 |
| POX | 7 | 82 | 22 |

**CLV** - Clevage site, **LIG** - Ligand Binding Site, **MOD** - Post-Translational Modification Site

**Table S3.2**: ELM patterns observed in the motifs obtained from Subcellular Localization dataset at a selection threshold of 9

| Class | CLV | LIG | MOD | Average (%)of ELM patterns |
| --- | --- | --- | --- | --- |
| CYT | 7 | 86 | 23 | 90.4 |
| CSK | 7 | 47 | 17 | 78.3 |
| END | 7 | 82 | 23 | 95.1 |
| EXC | 7 | 89 | 26 | 96.6 |
| GOL | 7 | 42 | 20 | 77.3 |
| LYS | 5 | 39 | 16 | 66.3 |
| MIT | 7 | 80 | 22 | 89.4 |
| NUC | 8 | 91 | 24 | 97.3 |
| PLA | 8 | 95 | 25 | 98.9 |
| POX | 6 | 48 | 20 | 78.3 |

**Table S4**: Prosite patterns of 50 enzyme families

| **Serial No.** | **Prosite Family** | **# of sequences** | **PERL RegEx pattern** |
| --- | --- | --- | --- |
| **1** | PS00056 | 795 | GD.[LIV].[LIVA].[QEK].[RK]P[LIV]S |
| **2** | PS00057 | 907 | [IVRLP][DYN][YLF].{2,3}[LIVMTPFS].{2}[LIVM].{2}[FYTS][LIVMT][STNQG][DERPN].{1,2}[GYAH][KCR][LIVM].{3}[RHG][LIVMASR] |
| **3** | PS00061 | 850 | [LIVSPADNK].{9}[^P].{2}Y[PSTAGNCV][STAGNQCIVM][STAGC]K[^PC][SAGFYR][LIVMSTAGD].[^K][LIVMFYW][^D].[^YR][LIVMFYWGAPTHQ][GSACQRHM] |
| **4** | PS00092 | 870 | [LIVMAC][LIVFYWA][^DYP][DN]PP[FYW] |
| **5** | PS00096 | 824 | [DEQHY][LIVMFYA].[GSTMVA][GSTAV][ST][STVM][HQ]K[STG][LFMI].[GAS][PGAC][RQ][GSARH][GA] |
| **6** | PS00111 | 721 | [KRHGTCVN][VT][LIVMF][LIVMC]R.D.N[SACV]P |
| **7** | PS00113 | 841 | [LIVMFYWCA][LIVMFYW]{2}DG[FYI]PR.{3}[NQ] |
| **8** | PS00153 | 772 | [IV]T.E.{2}[DE].{3}GA.[SAKR] |
| **9** | PS00163 | 803 | GS.{2}M.[^RS]K.N |
| **10** | PS00164 | 908 | [LIVTMS][LIVP][LIV][KQ].[ND]Q[INV][GA][ST][LIVM][STL][DERKAQG][STA] |
| **11** | PS00358 | 873 | [LIVM].{2}[LIVM][STAVC][GE][QV].{2}[LIVMA].[STC].[STAG][KRH].[STA] |
| **12** | PS00359 | 882 | [RKN].[LIVM].G[ST].{2}[SNQ][LIVM]G.[^M][LIVM].{0,1}[DENG] |
| **13** | PS00361 | 880 | [AV].{3}[GDNSR][LIVMSTAG].{3}GP[LIVM].[LIVM]PT |
| **14** | PS00376 | 732 | [GN][AS]GDQG.{3}G[FYHG] |
| **15** | PS00389 | 833 | [LIVM].[LIVMFYT].{3}[LIVMT][DENQK].[^G][LIVM].[GSA]G[LIVMFYGA][^S][LIVM][KRHENQ].[GSEN] |
| **16** | PS00449 | 776 | [STAGN][^E][STAG][LIVMF]RL[^LP][SAGV]N[LIVMT] |
| **17** | PS00455 | 733 | [LIVMFY][^E][^VES][STG][STAG]G[ST][STEI][SG].[PASLIVM][KR] |
| **18** | PS00469 | 752 | N.{2}H[GA]SD[GSA][LIVMPKNE] |
| **19** | PS00474 | 864 | [FL].{6}[DN].{2}[AGS].[ST].G[KRH]G.{2}G.{3}R |
| **20** | PS00475 | 838 | [KR][LIVM]{2}[GASL].[GT].[LIVMA].{2,5}[LIVMF].[LIVMF].{3,4}[LIVMFCA][ST].{2}A.{3}[LIVM].{3}G |
| **21** | PS00486 | 812 | [STA][LIVMF].[LIVM].DE[LIVMFY][GCA][RKHAS][GS][GST].{4}G |
| **22** | PS00579 | 776 | [KNQS][PSTLNH][^D][^F][LIVMFA][KRGSADN].[LIVYSTA][KR][KRHQS][DESTANQRL][LIV]A[KRCQVT][LIVMA] |
| **23** | PS00595 | 726 | [LIVFYCHT][DGH][LIVMFYAC][LIVMFYA].{2}[GSTAC][GSTA][HQR]K.{4,6}G.[GSAT].[LIVMFYSAC] |
| **24** | PS00646 | 856 | [KRQSEAT][GS].RH.{2}[GSNHKLCD].{2}[LIVMCT][RNH]GQ |
| **25** | PS00651 | 799 | G.{2}[GNF].{4}[VAI].{2}G[FY].{2}[NH][FYWL]L.{5}[GA].{3}[STNG] |
| **26** | PS00710 | 780 | [GSA][LIVMF].[LIVM][ST][PGA]SH[NIC]P |
| **27** | PS00732 | 859 | [LIVMT].[LIVM][KR]L[STAK]R[^E]G[AKR] |
| **28** | PS00745 | 953 | [ARH][STA].G.GGQ[HNGCSY][VI]N.{3}[ST][AKG][IV] |
| **29** | PS00828 | 872 | [CHDS].{2}[CND].{2}[LIVM].R.{3}[LIVMNR].[LIVM].[CN].{3,4}[KRSN][HLFR].[QCAV].Q |
| **30** | PS00831 | 805 | G.[LIVM]{2}.RQRG.{5}G |
| **31** | PS00893 | 773 | G.{5}E.{4}[TAGCV][LIVMACF].R[EL][LIVMFGSTA].[EA]E.[GNDTHR] |
| **32** | PS00905 | 796 | D[LIVMA]PG[LIVM]{2}[DEYPKQV][GN]A.{2}G.G |
| **33** | PS00936 | 777 | K[STNV][^F].[GSAM][SAILV].[KRA]R[IVFY].{14,16}[GSANQKR]H |
| **34** | PS00937 | 938 | K.{3}[KRCV].[LIVM]W[IVN][STNALVQCMI][RH][LIVM][NS].{3}[RKHSG] |
| **35** | PS00943 | 788 | N.{3}[DEH].{2}[LIMFYT]D.{2}[VM].R[ST].{2}R.{4}[GYNKR] |
| **36** | PS01015 | 782 | [LIVMF].[KRGTIEQSN].[GSAIYN][KRQDAVLSIH][VGAIT][RSNAK].{0,1}[KRAQ][SAKG][KYR][KLI][LYSFT][YF][LIM][RK] |
| **37** | PS01016 | 760 | [KRC][GSAT].{4}[FYWLMH][DQNGKRH].P.[LIVMFY].{3}H.{2}[GSA]H[LIVMFA] |
| **38** | PS01055 | 874 | K[LIVMF]DG[LIVMAS][SAG].{4}Y.{2}[GRD].[LF].{4}[ST]RG[DN]G.{2}G[DE][DENL] |
| **39** | PS01108 | 903 | [GDEN]D.[IV].[IV][LIVMA].G.{2}[KRA][GNQK].{2,3}[GA].[IV] |
| **40** | PS01131 | 738 | [LIVMAC][LIVMFYWT][DE].G[STAPVLCG]G.[GAS].[LIVMF][ST].{2,3}[LIVMA].{5,8}[LIVMYF].[STAGVLC][LIVMFYHCS]E.D |
| **41** | PS01143 | 800 | [DES][IVT].{4}H[PT][FAVY][FYW][TISN].{9,13}[GN][KRHNQ] |
| **42** | PS01166 | 941 | G.[KN][LIVMFA][STAC][GSTNR].[HSTA][GSAI][QNH]K[GL][IVTEC] |
| **43** | PS01176 | 812 | [GLES].[LIVM].{2}L[KR][KRHNS].K.{5}[LIVM].{2}[GNKADS].[DEN][CRG][GI] |
| **44** | PS01195 | 723 | [FYH].{2}[TN][RK]HN.G.{2}[LIVMFAYCT][LIVMFA][DEN] |
| **45** | PS01199 | 886 | [IMGV].{2}[LIVA].{2,3}[LIVMY][GAS].{2}[LMSF][GSNH][PTKR][KRAVG][GN].[LIMF]P[DENSTKQPRAGVI] |
| **46** | PS01275 | 828 | K.[AV].{4}G.{2}[LIVT].VP.{2}[LIVC].{2}[GD] |
| **47** | PS01280 | 888 | [GSA][PTAV].[YH]CPS[LIVMF][ED].K[LIVMFA].[KRNT][FY] |
| **48** | PS01312 | 812 | [IV].[IV][SA]T[NQ]MAGRG.DI.L |
| **49** | PS01319 | 734 | [LIVMF][QKRHSA][^E].[LIVMAC].{5,6}[LIVMW][RKAYF].[STACIVMF][PV][^LG][LIVMF].[FYI].{2}D |
| **50** | PS01348 | 720 | [NHS].{2}[NK].[TINAS][DN]G[ILVM]DG[LM] |

**Table S5**: Mapping and merging of discriminative *n­-*grams

| For sequence # 16 in GOL  >S35A3_HUMAN  MFANLKYVSLGILVFQTTSLVLTMRYSRTLKEEGPRYLSSTAVVVAELLKIMACILLVYKDSKCSLRALNRVLHDEILNKPMETLKLAIPSGIYTLQNNLLY**VALSNLDAA**T**YQVTYQLK**ILTTALFSVSMLSKKLGVYQWLSLVILMTGVAFVQWPSDSQLDSKELSAGSQFVGLMAVLTACFSSGFAGVYFEKILKETKQSVWIRNIQLGFFGSIFGLMGVYIYDGELVSKNGFFQGYNRLTWIVVVLQALGGLVIAAVIKYADNILKGFATSLSIILSTLISYFWLQDFVPTSVFFLGAILVITATFLYGYDPKPAGNPTKA  ---------------------------------------Initial mapping---------------------------------------  N-gram Sequence # Start Position End Position  ALSNLDAA 16 103 110  VALSNLDA 16 102 109  YQVTYQLK 16 112 119  -----------------------Sort n-grams based on start position--------------------------------  VALSNLDA, 16, 102, 109,  ALSNLDAA, 16, 103, 110,  YQVTYQLK, 16, 112, 119,  -------------------------Begin merging--------------------------------------------------------  Start Position End Position  102 110  Merged Gram :: **VALSNLDAA**  Start Position End Position  112 119  Merged Gram :: **YQVTYQLK** |
| --- |
