# Supplementary material for: Mining for class-specific motifs in protein sequence classification
Source: BMC Bioinformatics. 2013 Mar 15;14:96. doi: 10.1186/1471-2105-14-96 (PMC3610217; doi:10.1186/1471-2105-14-96)
Supplement: Additional file 2 — This file contains source code for six PERL modules used in this project. A full description of each program, usage and source code are provided in the README section at the beginning of the file. [file 1471-2105-14-96-S2.docx]

**Title: Mining for class-specific motifs in protein sequence classification**

This document includes the ReadMe file and the perl scripts that are part of the PERL Module.

**ReadMe file**

-------------------------------------------------------------------------------------------------------------------------------

*Authors*: Satish Mahadevan Srinivasan, Chittibabu Guda

The University of Nebraska Medical Center.

This program is free software: you can redistribute it and/or modify it under the terms of the GNU General Public License as published by the Free Software Foundation, either version 3 of the License, or (at your option) any later version. This program is distributed in the hope that it will be useful, but WITHOUT ANY WARRANTY; without even the implied warranty of MERCHANTABILITY or FITNESS FOR A PARTICULAR PURPOSE. See the GNU General Public License for more details. You should have received a copy of the GNU General Public License along with this program. If not, see http://www.gnu.org/licenses/

-------------------------------------------------------------------------------------------------------------------------------

Before using the PERL module we recommend users to go through this README file and get an understanding of the usage of the PERL scripts in this module.

This module includes 6 perl scripts. Description of each script is provided below:

*Substringremoval.pl*

This script removes all the short length discriminative n-grams that are sub-strings of longer length discriminative n-grams.

*Usage*: perl Substringremoval.pl <List_of_discriminative_n-grams_file>

*map_n_merge.pl*

This script will take two files as input. The first file should include Fasta sequences belonging to any ONE of the ten locations **X** (CYT, CSK, GOL, LYS, MIT, NUC, PLA, POX, END, and EXC) in a cell and the second file will have a list of discriminant n-grams from the corresponding location in the cell.

*Before using this script make sure that the shorter length discriminative n-grams that are sub-strings of longer length n-grams have been removed from the list of discriminant n-grams. To remove sub-strings from the discriminant n-gram list use the perl script Substringremoval.pl. This program may not work properly if the list of discriminant n-grams includes sub-string of a longer length discriminative n-gram.*

*Usage*: perl map_n_merge.pl <**X**_Fasta_sequence_file> <**X**_discriminant_ngrams_file>

*convert_prosite_pattern_toregex.pl*

This script converts PROSITE patterns to PERL RegEx patterns so that patterns can be searched in Fasta sequences using PERL scripts.

*Usage*: perl convert_prosite_pattern_toregex.pl <PROSITE_patterns_file>

*search_NLSdb_pattern.pl*

This script can be used for searching NLSdb patterns in the Fasta sequences of NUC (Nucleus) location. This script can also be used for searching NLSdb patterns in other locations of the cell.

If you obtain NLSdb patterns from <http://rostlab.org/services/nlsdb/browse.php> then convert all 'x' to '.' in NLSdb patterns.

*Usage*: perl search_NLSdb_pattern.pl <Fasta_sequence_file> <NLSdb_pattern_file>

*search_pattern_sequence.pl*

This script will help in searching for patterns in the Fasta sequences of different locations. For example let us consider a scenario where we want to look for Ligand Binding Sites (LIG) in Fasta sequences of NUC, CSK and/or any other cell location. The LIG patterns can be obtained from Eukaryotic Linear Motif (ELM) web site http://elm.eu.org/elms/browse_elms.html

*Usage*: perl search_pattern_sequence.pl <pattern_list_file> <Fasta_sequence_file>

*search_pattern_discriminative_ngrams.pl*

This script will help in searching for patterns in the discriminative n-grams. Since the discriminative n-grams will have only those patterns that are found in the Fasta sequences of any given location, this script will select fewer number of patterns from a list of patterns that are present in Fasta sequences and look for those selected patterns in the discriminative n-grams.

*Usage*: perl search_pattern_discriminative_ngrams.pl <pattern_list_file> <Fasta_sequence_file> <discriminative_ngrams_file>

*Substringremoval.pl*

#!/usr/bin/perl -w

#-----------------------------------------------------------------------------------------------------------------------------------------------

# Authors: Satish Mahadevan Srinivasan, Chittibabu Guda

# The University of Nebraska Medical Center.

# This program is free software: you can redistribute it and/or modify it under the terms of the GNU General Public License as published by the Free Software Foundation, either version 3 of the License, or (at your option) any later version. This program is distributed in the hope that it will be useful, but WITHOUT ANY WARRANTY; #without even the implied warranty of MERCHANTABILITY or FITNESS FOR A PARTICULAR PURPOSE. See the GNU General Public License for more details. You should have received a copy of the GNU General Public License along with this program. If not, see http://www.gnu.org/licenses/

# First created: 07/22/11

# Last modified: 10/28/12

#-----------------------------------------------------------------------------------------------------------------------------------------------

if ($#ARGV != 0) { print STDERR "---------------------------------------------------------

*Usage*: perl Substringremoval.pl <List_of_discriminative_n-grams_file>

---------------------------------------------------------\n";

exit;

}

my $TxtFile = shift; # This input file will contain all strings from which sub-string removal will be performed and the

# super-strings will be retained

open(IN,$TxtFile) || die "Cannot open the file: $TxtFile .\n";

# Declare two arrays for storing all the strings from the input file

my @Super_stringarray; # This array will hold the final list of super-strings.

my @AllStringarray; # This array will hold all the strings from the input file

# Take all the strings from input file and duplicate it in @Super_stringarray and @AllStringarray

while (<IN>)

{

my $string=$_; # Read each line from the input file

chomp $string;

push(@Super_stringarray,$string); # Add strings in to array

push(@AllStringarray,$string); # Add strings in to array

}

# Obtain the length of each array.

$Super_stringarray_length=scalar(@Super_stringarray);

$AllStringarray_length=scalar(@AllStringarray);

$initial_count=scalar(@Super_stringarray);

# Processing the array to remove sub-strings and retain the super-strings.

$Temp=-1;

l1: $Temp++;

for (; $Temp<$Super_stringarray_length;$Temp++) {

$Count=$Temp+1;

for (; $Count<$AllStringarray_length;$Count++) {

if (length $Super_stringarray[$Temp] < length $AllStringarray[$Count])

{

if (index($AllStringarray[$Count],$Super_stringarray[$Temp])>=0)

{

delete $Super_stringarray[$Temp];

delete $AllStringarray[$Temp];

$Super_stringarray_length=scalar(@Super_stringarray);

$AllStringarray_length=scalar(@AllStringarray);

goto l1;

}

else

{

next;

}

}

else

{

next;

}

}

}

# Remove any empty lines from the array

@Super_stringarray = grep(/\S/, @Super_stringarray);

# Obtain the count of number of super-strings obtained from the input file

$N_O_SS=scalar(@Super_stringarray);

print "Number of Super-Strings present in this file is :: $N_O_SS";

print "\n";

# Print all the Super-Strings in the input file

for my $Super_String (@Super_stringarray) {

print "$Super_String\n";

}

# Print the statistics

print "###################################################################################\n";

print "Number of strings in the input file :: $initial_count\n";

print "Number of Super-Strings present in this file is :: $N_O_SS\n";

$num_removed=$initial_count- $N_O_SS;

print "Number of Sub-Strings removed from the input file :: $num_removed\n";

print "###################################################################################\n";

*map_n_merge.pl*

#!/usr/bin/perl -w

#-----------------------------------------------------------------------------------------------------------------------------------------------

# Authors: Satish Mahadevan Srinivasan, Chittibabu Guda

# The University of Nebraska Medical Center.

# This program is free software: you can redistribute it and/or modify it under the terms of the GNU General Public License as published by the Free Software Foundation, either version 3 of the License, or (at your option) any later version. This program is distributed in the hope that it will be useful, but WITHOUT ANY WARRANTY; without even the implied warranty of MERCHANTABILITY or FITNESS FOR A PARTICULAR PURPOSE. See the GNU General Public License for more details. You should have received a copy of the GNU General Public License along with this program. If not, see http://www.gnu.org/licenses/

# First created: 07/26/11

# Last modified: 10/28/12

#-----------------------------------------------------------------------------------------------------------------------------------------------

if ($#ARGV != 1) { print STDERR "---------------------------------------------------------

*Usage*: perl map_n_merge.pl <**X**_Fasta_sequence_file> <**X**_discriminant_ngrams_file>

---------------------------------------------------------\n";

exit;

}

# Read the sequence file to begin with

my $fasta_sequence_file=shift; # sequence file containing fasta sequences

open(IN,$fasta_sequence_file) || die "Cannot open the file: $fasta_sequence_file .\n";

my @sequence; my @header; # arrays to hold fasta sequence and fasta header

while (<IN>)

{

$line=$_;

chomp($line);

if ($line=~/>/) {

push(@header,$line);}

else {

push(@sequence,$line);}

}

close(IN);

# Now read the discriminative n-grams

my $discriminative_ngrams_file=shift; # File containing discriminative n-grams

open(IN,$discriminative_ngrams_file) || die "Cannot open the file: $discriminative_ngrams_file .\n";

my @discriminative_ngrams; # array to hold discriminative n-grams

while (<IN>)

{

$line=$_;

chomp($line);

push(@discriminative_ngrams,$line);

}

close(IN);

# Get the length of the Fasta header, sequence and the discriminative n-grams

$header=@header; $sequence=@sequence; $discriminative_ngrams=@discriminative_ngrams;

# Initialize the variables

my $count=0; my $start=0; my $end=0;

my @n_grams; my @seq_num; my @start; my @end;

# Now calculate the sequence number, starting and ending index of the discriminative n-grams in fasta sequences. Check for all the discriminative

# n-grams across all the fasta sequences

for ($var=0;$var<$sequence;$var++) {

print "For seq # $var\n";

$reference=$var;

print "\n";

print "------------------------- Initial mapping-------------------------------\n";

print "\n";

print "\tN-gram\tSeq #\tStart Pos\tEnd Pos\n";

$temp="temp.txt"; # create a temp file to hold intermediate results

open(OUT,">",$temp) || die "Cannot open the file: $temp .\n";

for ($var_1=0;$var_1<$discriminative_ngrams;$var_1++) {

if($sequence[$var]=~/$discriminative_ngrams[$var_1]/) {

$position=index($sequence[$var],$discriminative_ngrams[$var_1]);

$result=$position+length($discriminative_ngrams[$var_1])-1;

print OUT "$discriminative_ngrams[$var_1], $var, $position, $result \n";

print "\t$discriminative_ngrams[$var_1]\t$var\t$position\t$result\n";

}

}

$cmd="sort -t ',' -k 3,3 -n $temp > temp1.txt "; # sort the contents of the temp file in to temp1 file

$cmd1="rm $temp";

system($cmd);

system($cmd1);

print "--------------------------------Sort n-grams based on start position----------------------------------------\n";

open(IN,"temp1.txt")|| die "Cannot open the file: temp1.txt .\n";

while (<IN>)

{

$line=$_;

chomp($line);

print "$line\n";

}

close(IN);

print "-----------------------------------------End output----------------------------------------------------------\n";

print"\n";

print "-------------------------Begin merging--------------------------------\n";

open(IN,"temp1.txt") || die "Cannot open the file: temp1.txt \n";

while (<IN>)

{

$line=$_;

chomp($line);

$count=$count+1;

my @str=split(/,/,$line);

push(@n_grams,$str[0]);

push(@seq_num,$str[1]);

push(@start,$str[2]);

push(@end,$str[3]);

}

close(IN);

# Get the size of each array for further processing

$seq_num=@seq_num; $start=@start; $end=@end; $n_grams=@n_grams;

$SIZE=0;

for ($SIZE=0;$SIZE<$count;) {

$Temp_var=0;

$start_pos=$start[$SIZE];

$end_pos=$end[$SIZE];

$Temp_var=$Temp_var+1;

while ($SIZE+1<$count) {

if ($start[$SIZE+1]<=$end_pos) {

$end_pos=$end[$SIZE+1];

$Temp_var=$Temp_var+1;

$SIZE=$SIZE+1;

}

elsif ($start[$SIZE+1]>$end_pos && $start[$SIZE+1]-$end_pos == 1) {

$end_pos=$end[$SIZE+1];

$Temp_var=$Temp_var+1;

$SIZE=$SIZE+1;

}

else {

goto L1;

}}

L1: print"";

print "\tStart Pos End Pos \n";

print "\t$start_pos\t$end_pos\n";

print "Merged Gram ::\t";

print substr ($sequence[$var],$start_pos,$end_pos-$start_pos+1);

print "\n";

$SIZE=$SIZE+1;

}

## After processing is over delete all the used variables including the arrays

for ($var_2=0;$var_2<$count;$var_2++) {

delete(@seq_num[$var_2]); delete(@start[$var_2]); delete(@end[$var_2]); delete(@n_grams[$var_2]);

}

## Modify the variables to hold a '0'

$count=0; $SIZE=0; $start_pos=0; $end_pos=0;

# Add a new line and delete the temporary files from the directory

print "\n";

$cmd3="rm temp1.txt"; system($cmd3);

}

*convert_prosite_pattern_toregex.pl*

#!/usr/bin/perl -w

#-----------------------------------------------------------------------------------------------------------------------------------------------

# Authors: Satish Mahadevan Srinivasan, Chittibabu Guda

# The University of Nebraska Medical Center.

# This program is free software: you can redistribute it and/or modify it under the terms of the GNU General Public License as published by the Free Software Foundation, either version 3 of the License, or (at your option) any later version. This program is distributed in the hope that it will be useful, but WITHOUT ANY WARRANTY; without even the implied warranty of MERCHANTABILITY or FITNESS FOR A PARTICULAR PURPOSE. See the GNU General Public License for more details. You should have received a copy of the GNU General Public License along with this program. If not, see http://www.gnu.org/licenses/

# First created: 08/30/11

# Last modified: 10/28/12

# This script uses the module PROSITE_2_regexp() developed by James Tisdall, 2001.

#-----------------------------------------------------------------------------------------------------------------------------------------------

#####################################################################################################################

# The PROSITE patterns in the file should be in the following format

# PA N-{P}-[ST]-{P}.

# PA [RK](2)-x-[ST].

# PA [ST]-x-[RK].

# PA [ST]-x(2)-[DE].

# PA [RK]-x(2,3)-[DE]-x(2,3)-Y.

# PA G-{EDRKHPFYW}-x(2)-[STAGCN]-{P}.

#####################################################################################################################

if ($#ARGV != 0) { print STDERR "---------------------------------------------------------

*Usage*: perl convert_prosite_pattern_toregex.pl <PROSITE_patterns_file>

---------------------------------------------------------\n";

exit;

}

# Take a list of PROSITE patterns available in a file

my $prosite_patterns_file=shift;

open (IN,$prosite_patterns_file) || die "Cannot open the file: $prosite_patterns_file .\n";;

while (<IN>) {

$line=$_;

chomp($line);

$line =~ s/PA //g;

print PROSITE_2_regexp($line);

print"\n";

}

close(IN);

#####################################################################################################################

#!/usr/bin/perl

#

##################################################

# parse_prosite:

#

# Parse patterns from the PROSITE database, and

# search for them in a protein sequence

#

# Copyright (c) 2001 James Tisdall

#

### Published November 2001

### Revision 20011228

# - Fixed bug in "join" of protein data that rearranged

# the lines of protein sequence (thanks to Brian Higgins)

# - Amended subroutine PROSITE_2_regexp to handle unusual [G>]

# in pattern of two PROSITE records (thanks to Andrew Dalke)

# - Amended subroutine PROSITE_2_regexp to handle

# unusual lowercase 'c' in one PROSITE record

# - Amended "get_line_types" subroutine and main program to

# handle multiple PA lines of pattern specification.

# - Amended subroutine PROSITE_2_regexp to handle

# long (>9) repeated elements

# - Added subroutine PROSITE_2_regexp_clever to demonstrate

# alternate method of translating (thanks to Andrew Dalke)

##################################################

sub PROSITE_2_regexp {

#

# Collect the PROSITE pattern

#

# my($pattern) = @_;

my $length=0;

#

# Copy the pattern to a regular expression

#

# my $regexp = $pattern;

my $regexp=$_[0];

#

# Now start translating the pattern to an

# equivalent regular expression

#

#

# Remove the period at the end of the pattern

#

$regexp =~ s/.$//;

#

# Replace 'x' with a dot '.'

#

$regexp =~ s/x/./g;

#

# Leave an ambiguity such as '[ALT]' as is.

# However, there are two patterns [G>] that need

# special treatment (and the PROSITE documentation

# is a bit vague, perhaps).

#

$regexp =~ s/\[G\>\]/(G|\$)/;

#

# Ambiguities such as {AM} translate to [^AM].

#

$regexp =~ s/{([A-Z]+)}/[^$1]/g;

#

# Remove the '-' between elements in a pattern

#

$regexp =~ s/-//g;

#

# Repetitions such as x(3) translate as x{3}

#

$regexp =~ s/\((\d+)\)/{$1}/g;

#

# Repetitions such as x(2,4) translate as x{2,4}

#

$regexp =~ s/\((\d+,\d+)\)/{$1}/g;

#

# '<' becomes '^' for "beginning of sequence"

#

$regexp =~ s/\</^/;

#

# '>' becomes '$' for "end of sequence"

#

$regexp =~ s/\>/\$/;

#

# Return the regular expression

#

return $regexp;

}

#####################################################################################################################

*search_NLSdb_pattern.pl*

#!/usr/bin/perl -w

#-----------------------------------------------------------------------------------------------------------------------------------------------

# Authors: Satish Mahadevan Srinivasan, Chittibabu Guda

# The University of Nebraska Medical Center.

# This program is free software: you can redistribute it and/or modify it under the terms of the GNU General Public License as published by the Free Software Foundation, either version 3 of the License, or (at your option) any later version. This program is distributed in the hope that it will be useful, but WITHOUT ANY WARRANTY; without even the implied warranty of MERCHANTABILITY or FITNESS FOR A PARTICULAR PURPOSE. See the GNU General Public License for more details. You should have received a copy of the GNU General Public License along with this program. If not, see http://www.gnu.org/licenses/

# First created: 09/01/11

# Last modified: 10/28/12

#-----------------------------------------------------------------------------------------------------------------------------------------------

if ($#ARGV != 1) { print STDERR "---------------------------------------------------------

*Usage*: perl search_NLSdb_pattern.pl <Fasta_sequence_file> <NLSdb_pattern_file>

---------------------------------------------------------\n";

exit;

}

# Obtain the fasta sequences and NLSdb patterns from input files

my $fasta_sequences_file=shift; # Obtain the fasta sequences of NUC

my $NLSdb_patterns_file=shift; # enter the pattern

# store the fasta sequences and the NLSdb patterns in an array

my @fasta_sequences;

open (IN,$fasta_sequences_file) || die "Cannot open the file: $fasta_sequences_file .\n";

while (<IN>) {

$line=$_;

chomp($line);

if($line =~/>/) {

} # if its a fasta header omit it

else {

push (@fasta_sequences,$line);

}

}

close(IN);

my @NLSdb_pattern;

open (IN,$NLSdb_patterns_file) || die "Cannot open the file: $NLSdb_patterns_file .\n";

while (<IN>) {

$line=$_;

chomp($line);

push(@NLSdb_pattern,$line);

}

close(IN);

# Determine the number of fasta sequences in NUC and number of NLSdb pattern

$NLSdb_pattern=@NLSdb_pattern;

$fasta_sequences=@fasta_sequences;

$pattern_identified=0;

for ($var_1=0;$var_1<$NLSdb_pattern;$var_1++) {

$pattern_found=0;

for ($var_2=0;$var_2<$fasta_sequences;$var_2++) {

while ($fasta_sequences[$var_2]=~m/$NLSdb_pattern[$var_1]/g) {

$pattern_found++;

}

}

if ($pattern_found > 0) {

$pattern_identified=$pattern_identified+1;

print "$NLSdb_pattern[$var_1] found\n";

}

}

print "################################################################################################\n";

print "Total number of NLSdb patterns = $NLSdb_pattern\n";

print "Total number of pattern match found in NUC (Nucleus) sequences = $pattern_identified\n";

print "#################################################################################################\n";

*search_pattern_sequence.pl*

#!/usr/bin/perl -w

#-----------------------------------------------------------------------------------------------------------------------------------------------

# Authors: Satish Mahadevan Srinivasan, Chittibabu Guda

# The University of Nebraska Medical Center.

# This program is free software: you can redistribute it and/or modify it under the terms of the GNU General Public License as published by the Free Software Foundation, either version 3 of the License, or (at your option) any later version. This program is distributed in the hope that it will be useful, but WITHOUT ANY WARRANTY; without even the implied warranty of MERCHANTABILITY or FITNESS FOR A PARTICULAR PURPOSE. See the GNU General Public License for more details. You should have received a copy of the GNU General Public License along with this program. If not, see http://www.gnu.org/licenses/

# First created: 11/07/11

# Last modified: 10/28/12

#-----------------------------------------------------------------------------------------------------------------------------------------------

if ($#ARGV != 1) { print STDERR "--------------------------------------------

*Usage*: perl search_pattern_sequence.pl <pattern_list_file> <Fasta_sequence_file>

---------------------------------------------------------\n";

exit;

}

# Obtain the file names for patterns and fasta sequences

$pattern_input_file=shift;

$fasta_sequence_input_file=shift;

# Declare an array to hold the list of patterns

my @pattern_list;

open (IN,$pattern_input_file)|| die "Cannot open the file: $pattern_input_file .\n";

while (<IN>) {

$line=$_;

chomp($line);

push(@pattern_list,$line);

}

close(IN);

# Obtain a count of number of patterns in the pattern input file

$pattern_list=@pattern_list;

# Look for each patterns in the fasta sequences

$pattern_found=0;

for ($var_1=0;$var_1<$pattern_list;$var_1++) {

print "Looking for pattern $pattern_list[$var_1] ";

$pattern_count=0; # keeps track of number of times a pattern is encountered in the fasta sequences

open (IN,$fasta_sequence_input_file) || die "Cannot open the file: $fasta_sequence_input_file .\n";

while (<IN>) {

$line=$_;

chomp($line);

if ($line=~/>/) {

} # if fasta header is encountered do not search for pattern

if ($line=~/$pattern_list[$var_1]/){ $pattern_count++;

} # is a pattern is found

}

close (IN);

# if the pattern occurs in the fasta sequences declare the availability of the pattern

if ($pattern_count > 0) {

$pattern_found=$pattern_found+1;

print "exits\n";

}

else {

print "does not exists\n";

}

}

print "#############################################################################################\n";

print "The total number of pattern's in the file=$pattern_list\n";

print "The total number of pattern's found=$pattern_found\n";

print "##################################################################################################\n";

*search_pattern_discriminative_ngrams.pl*

#!/usr/bin/perl -w

#-----------------------------------------------------------------------------------------------------------------------------------------------

# Authors: Satish Mahadevan Srinivasan, Chittibabu Guda

# The University of Nebraska Medical Center.

# This program is free software: you can redistribute it and/or modify it under the terms of the GNU General Public License as published by the Free Software Foundation, either version 3 of the License, or (at your option) any later version. This program is distributed in the hope that it will be useful, but WITHOUT ANY WARRANTY; without even the implied warranty of MERCHANTABILITY or FITNESS FOR A PARTICULAR PURPOSE. See the GNU General Public License for more details.You should have received a copy of the GNU General Public License along with this program. If not, see http://www.gnu.org/licenses/

# First created: 10/23/11

# Last modified: 10/28/12

#-----------------------------------------------------------------------------------------------------------------------------------------------

if ($#ARGV != 2) { print STDERR "---------------------------------------------------------

*Usage*: perl search_pattern_discriminative_ngrams.pl <pattern_list_file> <Fasta_sequence_file> <discriminative_ngrams_file>

---------------------------------------------------------\n";

exit;

}

# Get the inputs from the file

$pattern_input_file=shift; # file containing the patterns to be searched in fasta sequences

$fasta_sequence_file=shift; # file containing fasta sequences

$discriminative_ngrams_file=shift; # file containing merged discriminative n-grams

# Obtain the patterns in an array

my @pattern_list;

open (IN,$pattern_input_file) || die "Cannot open the file: $pattern_input_file .\n";

while (<IN>) {

$line=$_;

chomp($line);

push(@pattern_list,$line);

}

close(IN);

# Obtain the count of number of patterns in the input file

$pattern_list=@pattern_list;

# Create an array to store patterns found in the fasta sequences

$pattern_found=0;

my @patterns_found_in_fasta_sequences;

for ($var_1=0;$var_1<$pattern_list;$var_1++) {

print "pattern $pattern_list[$var_1] ";

$pattern_count=0;

open (IN,$fasta_sequence_file) || die "Cannot open the file: $fasta_sequence_file .\n";

while (<IN>) {

$line=$_;

chomp($line);

if ($line=~/>/) {

} # If the line is a fasta header so not look for patterns

if ($line=~/$pattern_list[$var_1]/) {

$pattern_count=$pattern_count+1;

} # if pattern is found increment the count

}

close(IN);

# If the pattern is found in the fasta sequences add them as potential patterns

if ($pattern_count > 0) {

$pattern_found=$pattern_found+1;

print "exits\n";

push (@patterns_found_in_fasta_sequences,$pattern_list[$var_1]);

}

else {

print "does not exists\n";

}

}

# for each of these potential list of patterns see if these patterns are present in discriminative n-grams

$pattern_detected=0;

foreach $var_2 (@patterns_found_in_fasta_sequences) {

print "pattern $var_2 ";

$count_pattern=0;

open (IN,$discriminative_ngrams_file) || die "Cannot open the file: $discriminative_ngrams_file .\n";

while (<IN>) {

$line=$_;

chomp($line);

if ($line=~/$var_2/) {

$count_pattern=$count_pattern+1;

}

}

close(IN);

if ($count_pattern > 0) {

$pattern_detected=$pattern_detected+1;

print "exits\n";

}

else {

}

}

print "############################################################################################\n";

print "The total number of pattern's in the file= $pattern_list\n";

print "The total number of pattern's found in protein sequences= $pattern_found\n";

print "The total number of pattern's found in merged discriminative n-grams= $pattern_detected\n";

print "##############################################################################################\n";
